# Discovery from a large-scaled survey of *Trichoderma* in soil of China

**DOI:** 10.1038/s41598-017-07807-3

**Published:** 2017-08-22

**Authors:** Kai Chen, Wen-Ying Zhuang

**Affiliations:** 1 State Key Laboratory of Mycology, Institute of Microbiology, Chinese Academy of Sciences, Beijing, 100101 China; 2 University of Chinese Academy of Sciences, Beijing, 100049 China

## Abstract

The first large-scaled survey of soil-inhabiting *Trichoderma* is conducted in 23 provinces of China. Twenty-three new species belonging to the green-ascospored clades are discovered. Their phylogenetic positions are determined by sequence analyses of the combined partial sequences of translation elongation factor 1-alpha and the second largest RNA polymerase subunit encoding genes. Morphology and culture characteristics are observed, described and illustrated in detail. Distinctions between the new species and their close relatives are compared and discussed. They are named as: *T. aggregatum*, *T. alpinum*, *T. bannaense*, *T. breve*, *T. brevicrassum*, *T. byssinum*, *T. chlamydosporicum*, *T. concentricum*, *T. ganodermatis*, *T. hainanense*, *T. hengshanicum*, *T. hirsutum*, *T. hunanense*, *T. ingratum*, *T. liberatum*, *T. linzhiense*, *T. longisporum*, *T. polypori*, *T. pseudodensum*, *T. simplex*, *T. solum*, *T. undatipile* and *T. zayuense*.

## Introduction

The genus *Trichoderma* Pers. is widespread and can be easily found in soil, on decaying wood or on other fungi. Species of the genus are ecologically and economically important. For example, *T. reesei* E.G. Simmons is a well-known industrial cellulose producer^1^; some species, like *T. harzianum* Rifai and *T. virens* (J.H. Mill., Giddens & A.A. Foster) Arx, are widely used in biocontrol of plant pathogens^2^; and some others are reported to have the potential to remediate soil and water pollutions^3^. However, a few of them are indicated as causal agents of green mold disease in mushroom cultivation^4, 5^, and may cause human diseases^6^.

According to ascospore color, *Trichoderma* species are divided into two parts, i.e. species having green ascospores and that producing hyaline ascospores. *Trichoderma* species having green ascospores were first intensively studied by Chaverri and Samuels^7^. They described 40 species including 11 new ones. Jaklitsch^8^ investigated the European species and added nine species to the genus. Subsequently, more species were further found: *T. amazonicum*, *T. guizhouense*, *T. pseudogelatinosum*, *T. lycogaloides* and *T. sulawesensis* ^5, 9–11^, and *T. rosulatum*, *T. rufobrunneum* and *T. stipitatum* ^12^. Recently, Chaverri *et al*.^13^ described nine more species in their revisionary work on the *T. harzianum* complex. Jaklitsch and Voglmayr^14^ introduced seven additional taxa, and defined the species with green ascospores as Green-spored Clade which contains several subclades: Ceramicum, Chlorosporum, Haizianum, Helicum, Spinulosum and Strictipile, as well as scattered terminal branches. And their treatment has not been accepted by many authors.

Soil is an important substrate for *Trichoderma*. As indicated by Jaklitsch^8, 14^, studies focused on soil-inhabiting species of the genus have been carried out by different researchers around the world. In China, Wen *et al*.^15^ performed the first survey of *Trichoderma* in soil. Among the 301 strains from southwestern China, they identified nine species based on morphological characteristics. Later, Zhang *et al*.^16^ identified 11 species in 64 isolates from Hebei, Tibet, Yunnan and Zhenjiang provinces based on the combined analyses of morphology and molecular data. Sun *et al*.^17^ performed the most extensive biodiversity survey of soil-inhibiting *Trichoderma* species in China. They isolated 1910 strains from 20 provinces, and recognized 23 species based on morphological features and oligonucleotide barcode program (TrichOKEY v. 1.0 and TrichoBLAST). Most recently, three more species were added to the genus from Guizhou, Hubei and Tibet^11, 18^.

Although many studies have been focused on soil-inhabiting *Trichoderma* species, up to now approximately 50 species are reported from soil, which is obviously a small fraction of the known species in the genus. This situation may be due to (1) phenotypic characters alone or information provided by ITS sequences are insufficient for species identification; (2) the previous work has paid more attention to wood-inhabiting species^14, 19^, and (3) researchers concentrated on soil-inhabiting species were sampling in limited areas or farmlands, which leads to the deficiency of knowledge of species diversity. Therefore, a comprehensive survey is required to assess the biodiversity of *Trichoderma* in soil.

In the present study, we try to update our understanding the species diversity of *Trichoderma* from soil in China using an integrated study of morphology and molecular data. Among the 85 species identified (unpublished data), 23 new species belonging to the green-ascospored clades are here introduced.

## Results

### Phylogenetic analyses

**Figure 1:**
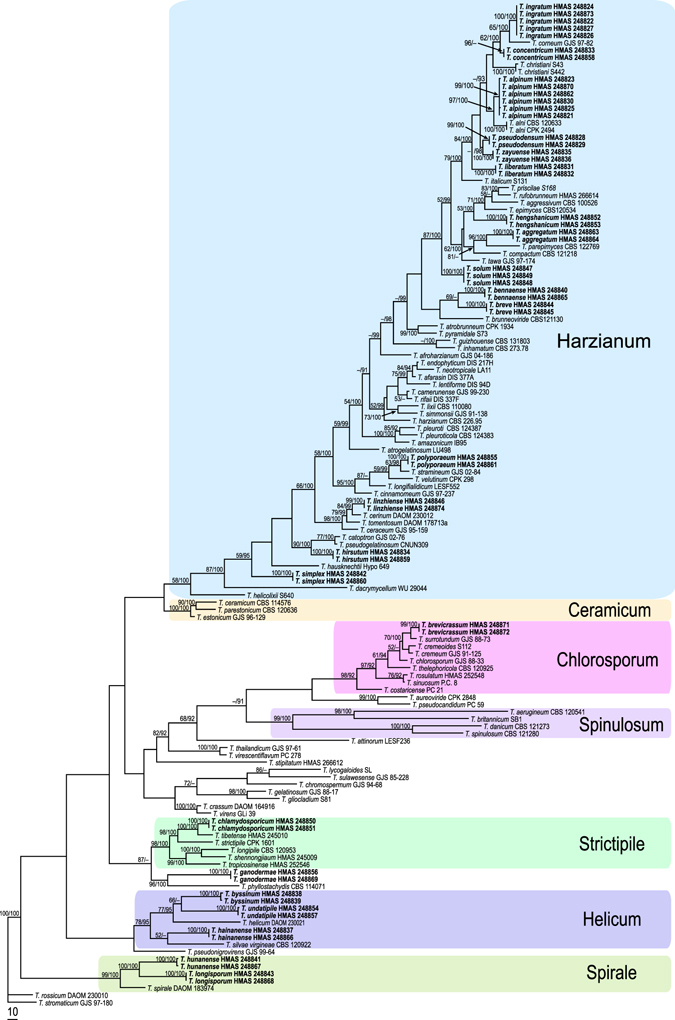
Phylogram generated from maximum parsimony analysis based on combined RPB2 and TEF1 sequence data of green-ascospored *Trichoderma* species with *T. rossicum* and *T. stromaticum* as outgroup taxa. MPBP above 50% (left) and BIPP above 90% (right) are indicated at nodes. New species proposed are indicated in boldface.

The partition homogeneity test (P = 0.01) indicated that the individual partitions were congruent thus RPB2 and TEF1 sequences were combined for analyses^20^. The combined sequence matrix contained 138 sequences (105 species) and 2271 characters (1053 for RPB2 and 1217 for TEF1). Of the characters included in the matrix, 743 were parsimony-informative, 1377 were constant, and 151 were parsimony-uninformative. Maximum parsimony (MP) analyses generated 56 most parsimonious trees with similar topology. One of them is shown in Fig. 1 (tree length = 5264; consistency index (CI) = 0.2838; homoplasy index (HI) = 0.7162; retention index (RI) = 0.7005; rescaled consistency index (RC) = 0.1988). Bayesian inference (BI) analyses generated a bayesian tree similar to the MP trees in topology with minor differences.

In the resulted tree (Fig. 1), 7 clades were recognized: Ceramicum, Chlorosporum, Haizianum, Helicum, Spinulosum, Spirale and Strictipile, among which the Spirale Clade is newly introduced. The 23 new species were either scattered among these clades or show as separate terminal branches. Fifteen of the new species were in the Harzianum Clade, three in the Helicum Clade, two in the Spirale Clade, one in the Chlorosporum Clade, one in the Strictipile Clade, and one did not belong to any of the named clades.

### Taxonomy

**Figure 2:**
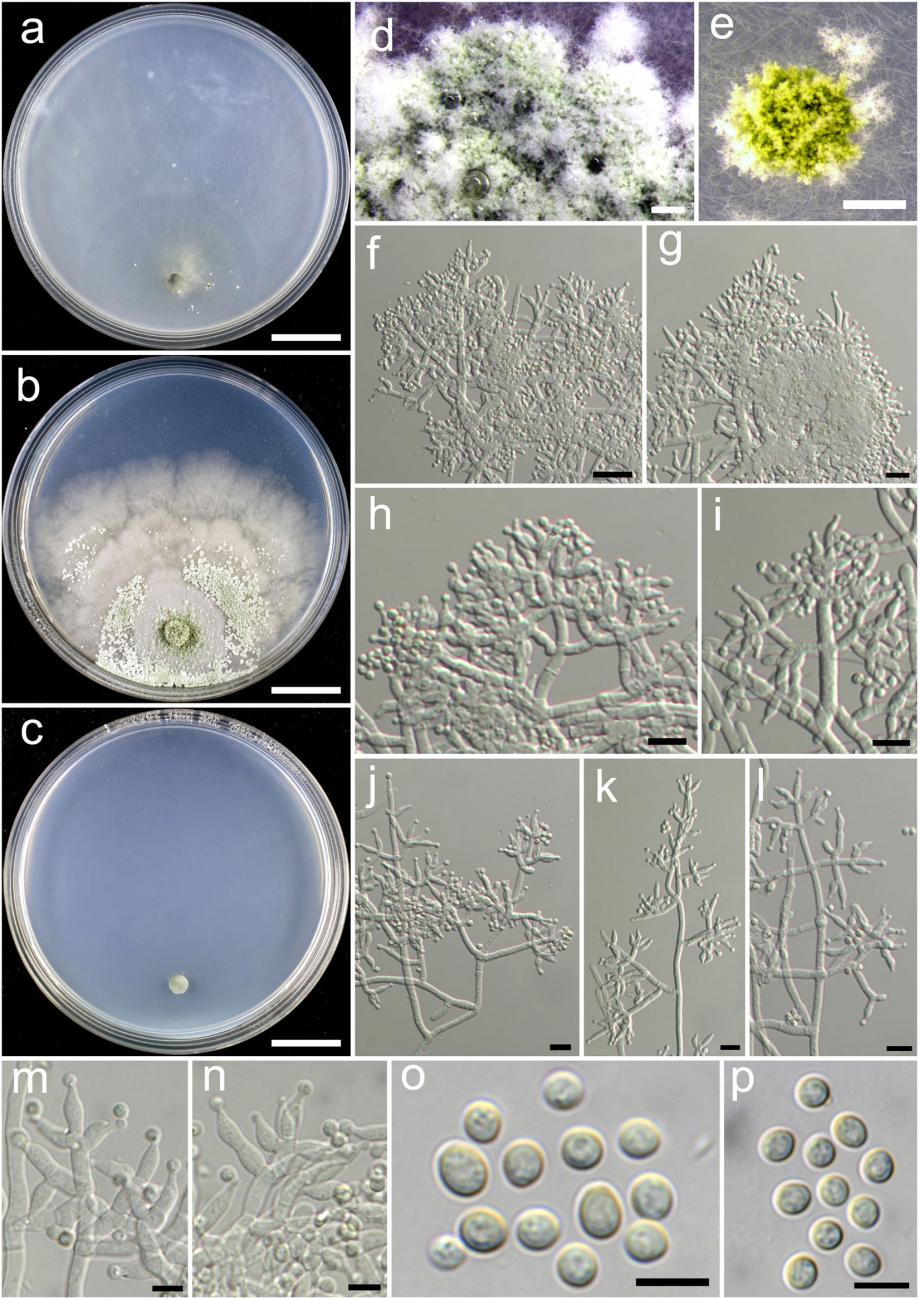
*Trichoderma aggregatum* (HMAS 248863). (**a**–**c**) Cultures at 25 °C (a. on CMD, 10 d; b. on PDA, 10 d; c. on SNA, 30 d); (**d**,**e**) Conidial pustules (d. PDA, 10 d; e. CMD, 10 d); (**f**–**n**) Conidiophores and phialides (PDA, 7 d); (**o**–**p**) Conidia (PDA, 7 d). Bars: a–c = 20 mm. d = 200 μm. e = 400 μm. f = 20 μm. j–l = 10 μm. m–p = 5 μm.

**Trichoderma aggregatum** K. Chen & W.Y. Zhuang, **sp. nov**. Figure 2

Fungal Names FN570406

*Etymology*: The epithet refers to the densely aggregated branches of conidiophores.

*Holotype*: CHINA, SICHUAN: Garze Tibetan Autonomous Prefecture, Luding County, elev. 2850 m, isolated from soil, September 2016, *K. Chen*, *TC927* (HMAS 248863). Ex-type culture CGMCC 3.18406.

On CMD after 72 h colony radius 24–25 mm, mycelium covering the plate after 8 d at 25 °C. Colony hyaline, radial, mycelium loose, aerial hyphae absent. Conidiation staring after 6 d, formed in pustules, pustules rare, spreading near the original inoculum, hemispherical, loose, white, turning green after 10 d. No chlamydospores observed. No distinct odour, no diffusing pigment observed.

On PDA after 72 h colony radius 12–16 mm, mycelium covering the plate after 13–14 d at 25 °C. Colony dense, margin slightly lobed, not well defined, aerial hyphae common. Conidiation starting after 5 d, formed on aerial hyphae or in pustules, pustules numerous, spreading circled around the original inoculum, pulvinate to hemispherical, loose, first discrete, turning aggregated, green. Conidiophores in pustules tree-like, branches densely disposed, paired or in whorls of 3, arising in acute angles or inclined upwards, rebranching 1–2 times. Conidiophores on aerial hyphae trichoderma-like, comprising a long main axis, short branches loosely disposed on it, solitary, paired or in whorls of 3, arising in straight or acute angle with the main axis, not or rebranching one time. Phialides numerous, typically formed in whorls of 3–4, ampulliform to lageniform, (6.4–)8.1–11.1(−13.9) × 2.5–3.3 μm, l/w 2.0–4.2, 1.4–2.8 μm wide at the base (n = 40). Conidia green, smooth, globose to subglobose, 2.5–3.9 × 2.5–3.1 μm, l/w 1.0–1.2(−1.3) (n = 40). No chlamydospores observed. No distinct odour, no diffusing pigment observed.

On SNA after 72 h colony radius 3–4 mm, mycelium covering the plate after 21 d at 25 °C. Colony hyaline, mycelium in distinct, aerial hyphae lacking. Conidiation not noted in 30 d. No chlamydospores observed. No distinct odour, no diffusing pigment observed.

*Additional strain examined*: CHINA, SICHUAN: Garze Tibetan Autonomous Prefecture, Luding County, elev. 2850 m, isolated from soil, September 2016, *K. Chen*, *TC928* (HMAS 248864).

*Notes*: Morphologically *T. aggregatum* is similar to *T. pseudodensum* in ampulliform to lageniform phialides that are densely disposed on conidiophores. However, *T. pseudodensum* differs in larger conidia and much higher growth rates on all of the three media. Phylogenetically, *T. aggregatum* is closely related to *T. parepimyces*. But the latter species produces larger conidia [(5–)6–10(−16) × (2.7–)3.0–3.7(−4.3) μm] and grows faster on PDA and SNA at 25 °C (22–24 mm on PDA and 24–26 mm on SNA after 3 d)^8^.

**Figure 3:**
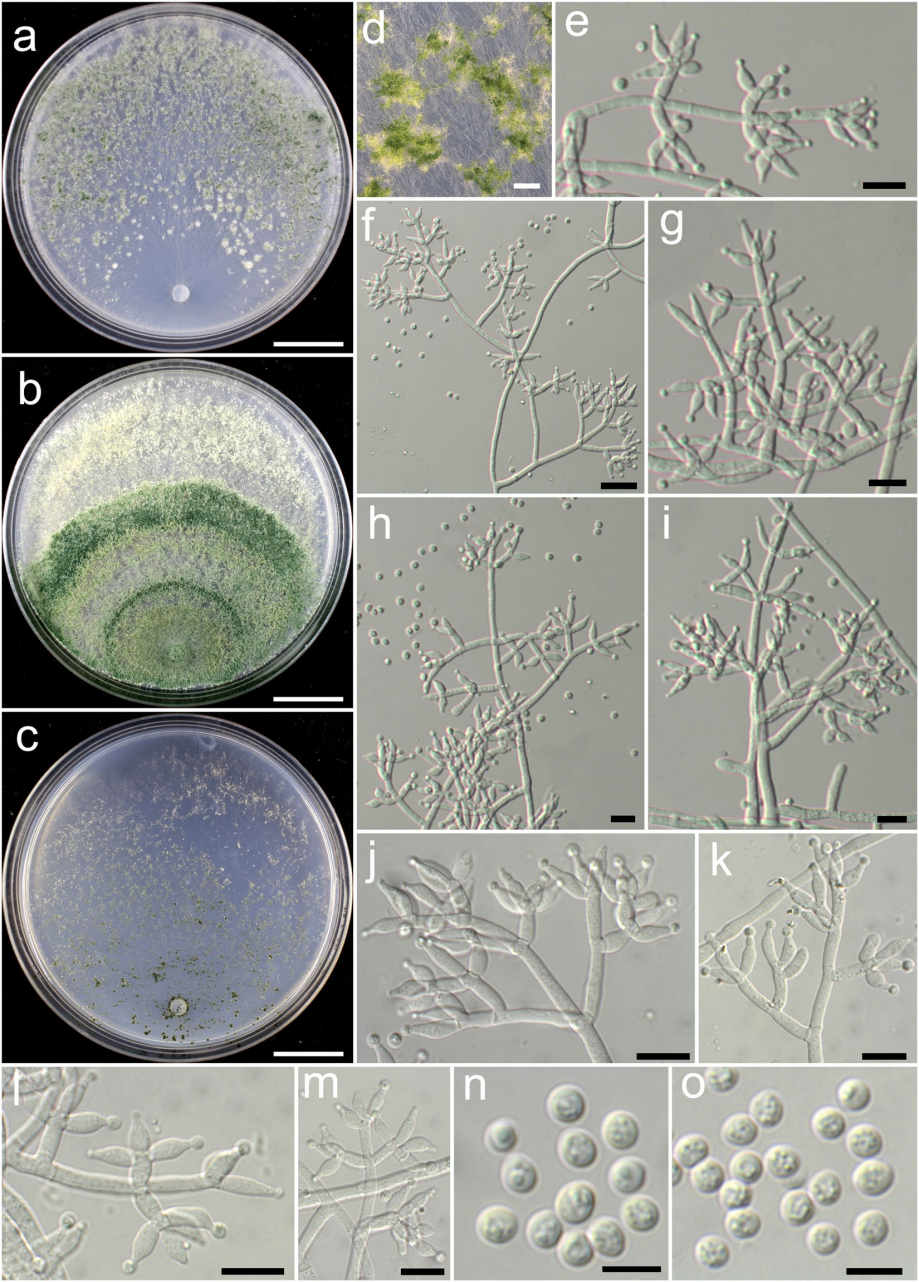
*Trichoderma alpinum* (HMAS 248821). (**a**–**c**) Cultures at 25 °C, 7 d (a. on CMD; b. on PDA; c. on SNA); (**d**) Conidial pustules (CMD, 7 d); (**e**–**m**) Conidiophores and phialides (PDA, 5 d); (**n**–**o**) Conidia (PDA, 5 d). Bars: a–c = 20 mm. d = 800 μm. f = 20 μm. e, g–m = 10 μm. n–o = 5 μm.

**Trichoderma alpinum** K. Chen & W.Y. Zhuang, **sp. nov**. Figure 3

Fungal Names: FN570405

*Etymology*: The specific epithet refers to the collected sites at a relatively high elevitude.

*Holotype*: CHINA, SICHUAN: Jiuzhaigou Valley National Nature Reserve, elev. 2300 m, isolated from soil, August 2014, *K. Chen*, *TC20* (HMAS 248821). Ex-type culture CGMCC 3.18385.

On CMD after 72 h colony radius 61–64 mm, mycelium covering the plate after 4 d at 25 °C. Colony hyaline, radial, not zonate, mycelium dense. Aerial hyphae common, more abundant in distant areas, absent in colony center. Conidiation starting after 3 d, effuse in aerial hyphae or in loose shrubs. No chlamydospores observed. No distinct odour, no diffusing pigment observed.

On PDA after 72 h colony radius 49–50 mm, mycelium covering the plate after 5 d at 25 °C. Colony dense, circular, not finely zonate. Aerial hyphae abundant, more abundant at colony center, becoming fertile. Conidiation starting after 2 d, effuse in aerial hyphae or small granules, granules more abundant in distant areas, turning green. Conidiophores numerous, trichoderma-like, branches paired or unpaired, at right or acute angles with the main axis, not or rebranching once. Phialides paired or in whorls of 3–4, typically lageniform, sometimes ampulliform, 6.2–11.1 × 2.4–4.2 μm, l/w (1.5–)2.0–3.5(−4.0), 1.4–3.3 μm wide at the base (n = 40). Conidia light green, smooth, with several minute guttules, globose to subglobose, 3.1–3.9(−4.2) × 2.8–3.5 μm, l/w 1.0–1.2 (n = 40). No chlamydospores observed. No distinct odour, no diffusing pigment observed.

On SNA after 72 h colony radius 28–30 mm and mycelium covering the plate after 7 d at 25 °C. Colony hyaline, mycelium loose, margin not well defined, aerial hyphae common. Conidiation starting after 2 d, effuse in aerial hyphae or in small granules, denser around the original inoculum. No chlamydospores observed. No distinct odour, no diffusing pigment observed.

*Additional strains examined*: CHINA, HUBEI: Shennongjia Natural Reserve, elev. 2300 m, isolated from soil, September 2014, *K. Chen*, *TC206* (HMAS 248825); *ibid*., *TC227* (HMAS 248830); SICHUAN: Ngawa, elev. 2300 m, isolated from soil, August 2014, *K. Chen*, *TC137* (HMAS 248823); SICHUAN: Ngawa, elev. 3200 m, isolated from soil, September 2016, *K. Chen*, *TC914* (HMAS 248862); TIBET: Linzhi, Milin County, elev. 3160 m, isolated from soil, September 2016, *K. Chen*, *TC956* (HMAS 248870).

*Notes*: The collecting sites of *T. alpinum* are all located at an elevitude above 2000 m, which indicates that the fungus might be adaptable to cool and mountainous areas. Morphologically *T. alpinum* is characterized by producing loose shrubs on CMD, lageniform phialides and conidia with several minute guttules. Phylogenetically, *T. alpinum* is closely related to *T. alni*, but the latter species differs in distributing in low-elevation, slower growth on CMD and PDA and smaller conidia (2.5–3.5 × 2.5–2.7 μm)^21^.

**Figure 4:**
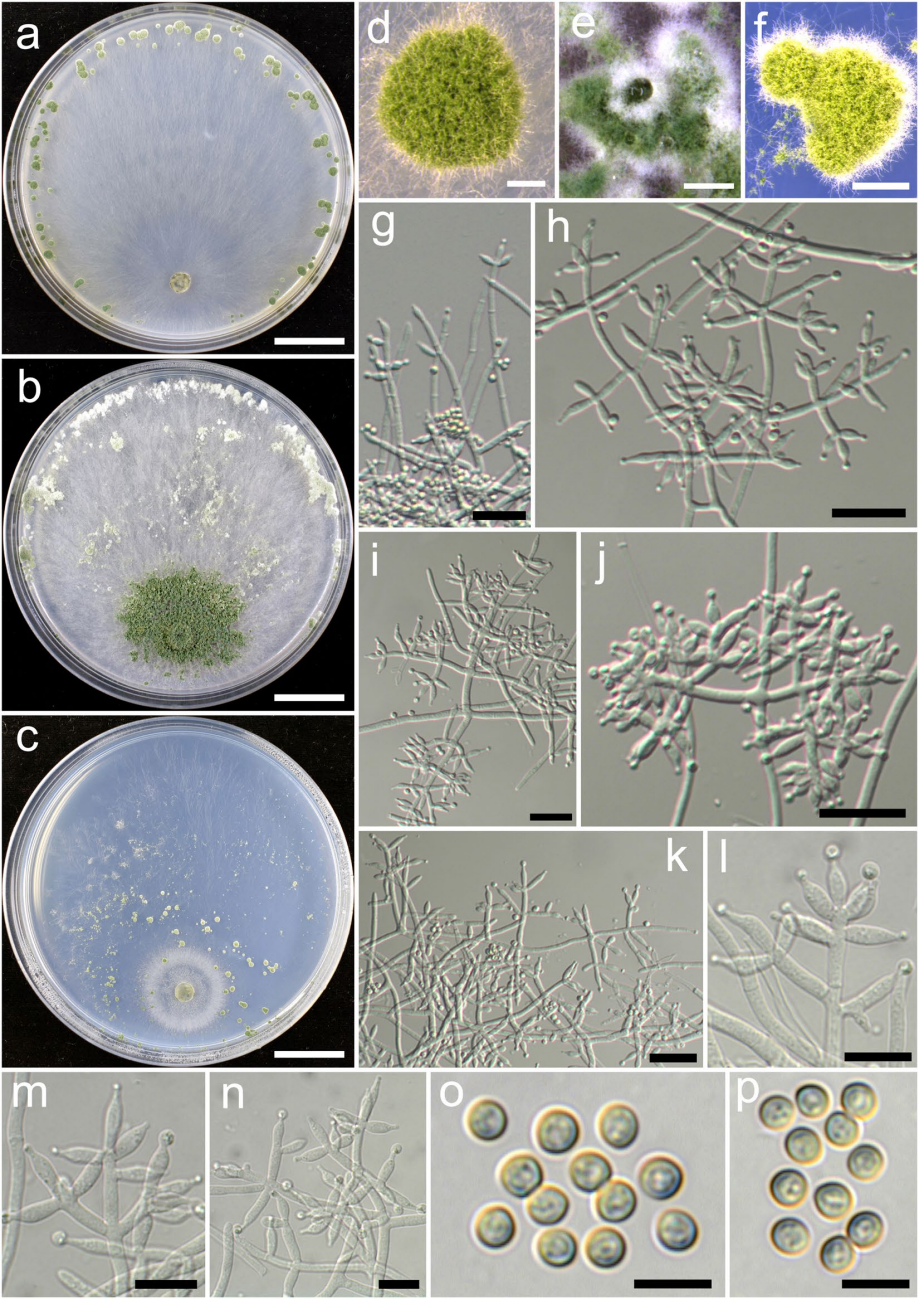
*Trichoderma bannaense* (HMAS 248840). (**a**–**c**) Cultures at 25 °C, 10 d (a. on CMD; b. on PDA; c. on SNA); (**d**–**f**) Conidial pustules (d. CMD, 7 d; e. PDA, 7 d; f. SNA, 7 d); (**g**–**n**) Conidiophores and phialides (g. SNA, 10 d; h–n. PDA, 3 d); (**o**–**p**) Conidia (PDA, 3 d). Bars: a–c = 20 mm. d = 250 μm. e = 400 μm. f = 800 μm. g–k = 20 μm. l–n = 10 μm. o–p = 5 μm.

**Trichoderma bannaense** K. Chen & W.Y. Zhuang, **sp. nov**. Figure 4

Fungal Names FN570408

*Etymology*: The specific epithet refers to the type locality Xishuangbanna.

*Holotype*: CHINA, YUNNAN: Xishuangbanna, elev. 800 m, isolated from soil, December 2014, *K. Chen*, *TC564* (HMAS 248840). Ex-type culture CGMCC 3.18394.

On CMD after 72 h colony radius 30–31 mm, mycelium covering the plate after 6 d at 25 °C. Colony hyaline, radial, mycelium loose, aerial hyphae nearly lacking. Conidial pustules formed after 4 d, common, spreading along the colony margin, hemispherical, compact, remaining discrete, 1–3 mm diam, first white, turning green after 5 d, with short hairs extending beyond the surface. No chlamydospores observed. No distinct odour, no diffusing pigment observed.

On PDA after 72 h colony radius 22–24 mm, mycelium covering the plate after 7 d at 25 °C. Colony hyaline, radial, mycelium dense, aerial hyphae common. Conidiation starting after 2 d, effused on aerial hyphae near the original inoculum. Conidial pustules noted after 4 d, first appearing around the original inoculum, then around the periphery, aggregated, with green drops on the surface, first white, turning green after 5 d. Conidiophores trichoderma-like, branches solitary or in whorls of 2–4, substituted by phialides at and near the tip, often arising in right angles with the main axis, not or rebranching one time. Phialides formed solitary or in whorls of 2–5, lageniform, sometimes ampulliform, (6.9–)7.5–10.0(−11.9) × 2.6–3.6 μm, l/w 2.1–3.9, 1.5–2.5 μm wide at the base (n = 40). Conidia green, smooth, globose to subglobose, sometimes ellipsoid, 2.5–3.6(−3.9) × 2.5–3.1 μm, l/w 1.0–1.2(−1.3) (n = 40). No chlamydospores observed. No distinct odour, no diffusing pigment observed.

On SNA after 72 h colony radius 2–5 mm, mycelium covering the plate after 10–12 d at 25 °C. Colony hyaline, margin irregular, not well defined, mycelium loose, aerial hyphae long, not common. Conidiation starting after 5 d, effused on short erect conidiophores and aerial hyphae near the original inoculum. Conidial pustules noted after 6 d, common, distributed around the colony center, hemispherical, remaining discrete, 0.5–1.5 mm diam, first white, turning green after 7 d, with hairs protruding beyond the surface, hairs straight, tips infrequently branched, fertile. No chlamydospores observed. No distinct odour, no diffusing pigment observed.

*Additional strain examined*: CHINA, YUNNAN: Xishuangbanna, elev. 800 m, isolated from soil, September 2016, *K. Chen*, *TC943* (HMAS 248865).

*Notes*: *Trichoderma bannaense* is distinctive by its elongated fertile tips of conidiophores. Although its greenish discrete pustules on CMD resembles those produced by *T. hamatum*, the latter has short and wide phialides^14^. Phylogenetically, *T. bannaense* is closely related to *T. breve* (see below for description and illustration), but the latter gives rise to numerous chlamydospores on PDA, and grows much faster on all three media.

**Figure 5:**
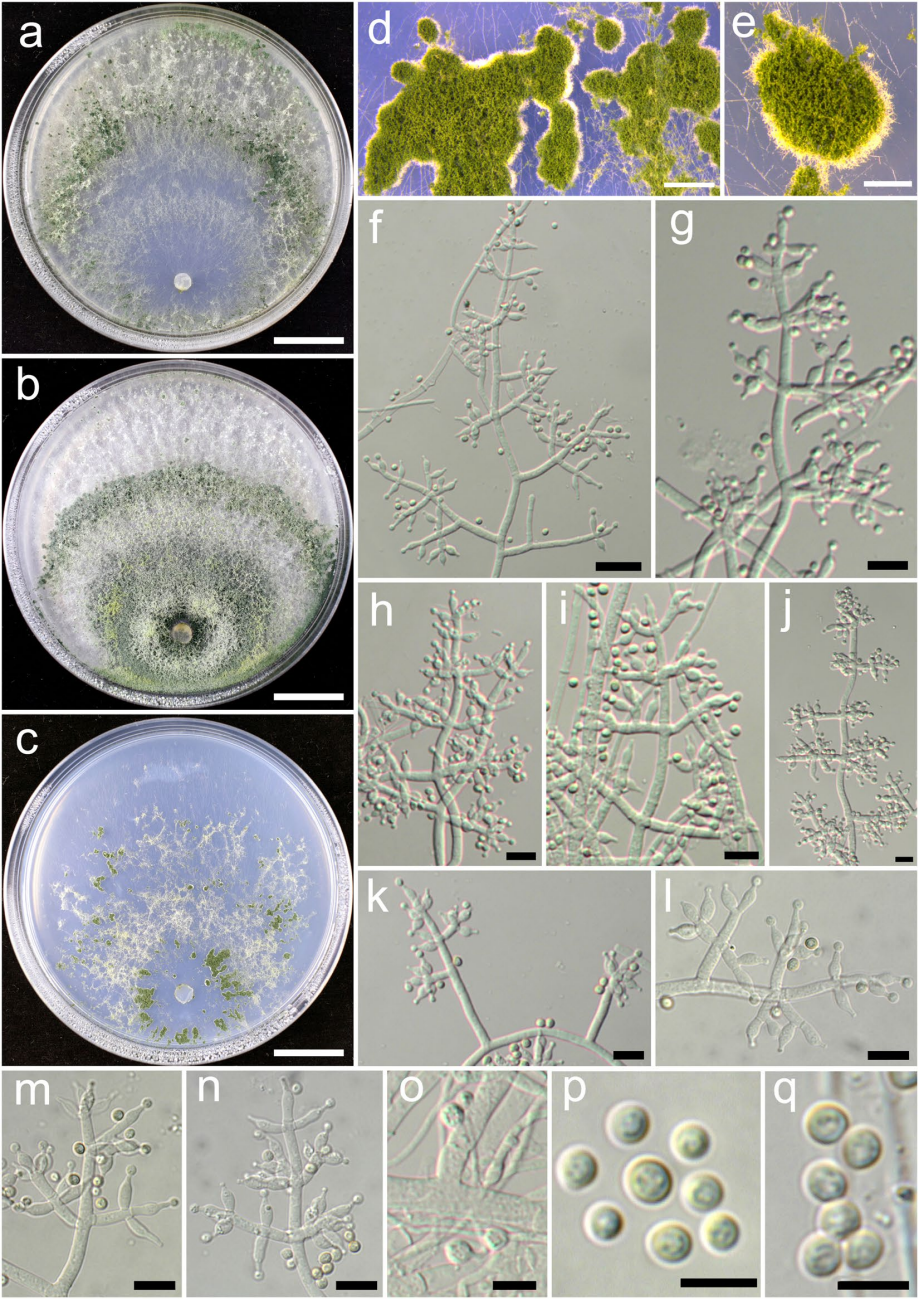
*Trichoderma breve* (HMAS 248844). (**a**–**c**) Cultures at 25 °C, 7 d (a. on CMD; b. on PDA; c. on SNA); (**d**,**e**) Conidial pustules (SNA, 7 d); (**f**–**n**) Conidiophores and phialides (f–j), (**l**–**n**). PDA, 2 d; (**k**). CMD, 2 d); (**o**) Chlamydospores (PDA, 2 d); (**p**–**q**) Conidia (PDA, 2 d). Bars: a–c = 20 mm. d = 400 μm. e = 200 μm. f = 20 μm. g–o = 10 μm. p–q = 5 μm.

**Trichoderma breve** K. Chen & W.Y. Zhuang, **sp. nov**. Figure 5

Fungal Names FN570404

*Etymology*: The specific epithet refers to the short phialides of the species.

*Holotype*: CHINA, BEIJING: Yanqing County, elev. 926 m, isolated from soil, July 2015, *K. Chen*, *TC735* (HMAS 248844). Ex-type culture CGMCC 3.18398.

On CMD after 72 h colony radius 67–72 mm, mycelium covering the plate after 3 d at 25 °C. Colony hyaline, radial, not finely zonate. Aerial hyphae abundant, long and wooly, spreading in 2–3 concentric zones, more abundant in distance areas. Conidiation starting after 2 d, effused on aerial hyphae, numerous. No chlamydospores observed. No distinct odour, no diffusing pigment observed.

On PDA after 72 h colony radius 71–72 mm, mycelium covering the plate after 3 d at 25 °C. Colony radial, zonate, mycelium dense, aerial hyphae abundant, long, spreading throughout the colony, forming a loose, floccose mat. Conidiation starting after 2 d, effused on aerial hyphae, numerous shrubs formed after 4 d, spreading in several concentric rings. Conidiophores symmetry, trichoderma-like, often with a long main axis up to 160 μm, side branches short, not or rebranching once. Phialides formed solitary, paired or in whorls of 3, ampulliform or lageniform, 6.7–10.0(−12.2) × 2.8–3.9 μm, l/w (1.7–)2.2–3.4(−4.4), 1.4–2.5 μm wide at the base (n = 40). Conidia green, smooth, globose to subglobose, 2.5–3.5(−3.9) × 2.5–3.1 μm, l/w 1.0–1.3 (n = 40). Chlamydospores common, globose or ellipsoid, 4.1–7.6 × (2.8–)3.5–6.2 μm, l/w 1.0–1.4(−1.8) (n = 20). No distinct odour, no diffusing pigment observed.

On SNA after 72 h colony radius 54–55 mm, mycelium covering the plate after 4 d at 25 °C. Colony similar to CMD, but with less aerial hyphae. Conidiation starting after 2 d, effused on aerial hyphae, conidial pustules formed after 4 d, common, spreading in irregular concentric rings, pulvinate or hemispherical, 1–5 mm diam, green. Hairs extending beyond the surface, short, straight or sinuous. Chlamydospores rare. No distinct odour, no diffusing pigment observed.

*Additional strain examined*: CHINA, BEIJING: Yanqing County, elev. 926 m, isolated from soil, July 2015, *K. Chen*, *TC736* (HMAS 248845).

*Notes*: *Trichoderma breve* is distinctive by the short and wide phialides. It is similar to the *T. harzianum* complex in colony morphology and relatively high growth rates. However, our phylogenetic analyses indicate *T. breve* is not associated with the *T. harzianum* complex, but closely related to *T. bannaense* (Fig. 1). The distinctions between the two species were already discussed.

**Figure 6:**
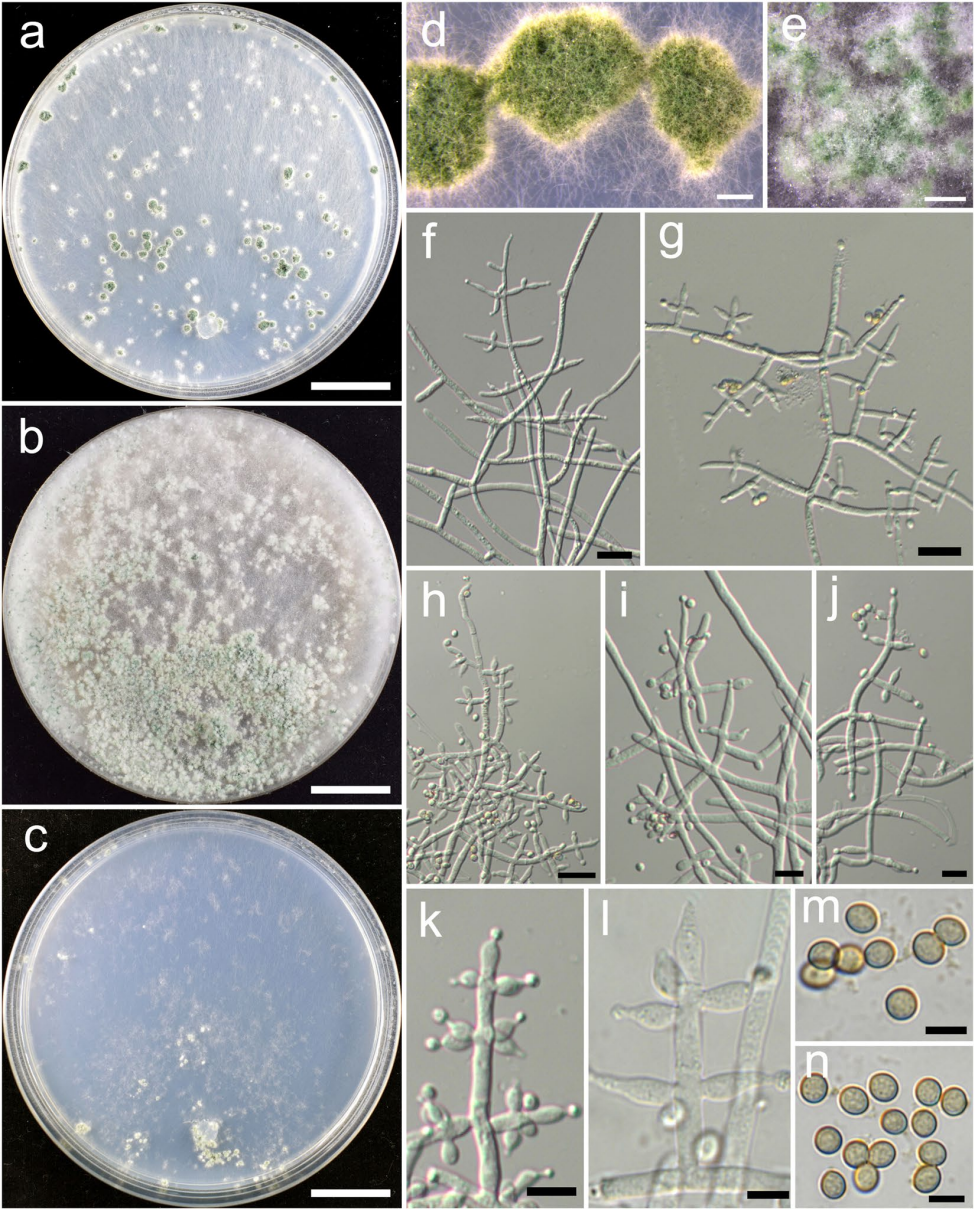
Trichoderma brevicrassum (HMAS 248871). (**a**–**c**) Cultures at 25 °C (a. on CMD, 10 d; b. on PDA, 10 d; c. on SNA, 15 d); (**d**–**e**) Conidial pustules (d. CMD, 10 d; e. PDA, 10 d); (**f**–**l**) Conidiophores and phialides (f–j, l. PDA, 4 d; k. CMD, 7 d); (**m**–**n**) Conidia (PDA, 4 d). Bars: a–c = 20 mm. d = 600 μm. e = 1 mm. f–h = 20 μm. i–k = 10 μm. l–n = 5 μm.

**Trichoderma brevicrassum** K. Chen & W.Y. Zhuang **sp. nov**. Figure 6

Fungal Names: FN570407

*Etymology*: The specific epithet refers to the short and wide phialides.

*Holotype*: CHINA, TIBET: Linzhi, Motuo County, elev. 1150 m, isolated from soil, September 2016, *K. Chen*, *TC967* (HMAS 248871). Ex-type culture CGMCC 3.18407.

On CMD after 72 h colony radius 63–66 mm, mycelium covering the plate after 3 d at 25 °C. Colony hyaline, radial, mycelium loose, aerial hyphae inconspicuous. Conidiation noted after 6 d, formed in pustules, pustules first formed along the colony margin, spreading throughout the colony, pulvinate, loose, 1–3.5 mm diam, first white, turning green after 7 d. No chlamydospores observed. No distinct odour, no diffusing pigment observed.

On PDA after 72 h colony radius 70–72 mm, mycelium covering the plate after 3 d at 25 °C. Colony dense, aerial hyphae abundant, wooly, long, extending up to the Petri dish cover. Conidiation starting after 3 d, formed on aerial hyphae and in shrubs, shrubs formed abundant on aerial hyphae, denser at the colony center, white, turning dark green after 5 d. Conidiophores asymmetry, irregularly branched, rebranching 1–3 times. Phialides typically formed in whorls of 3–4, not commonly paired or solitary, variable in shape and size, lageniform, ampulliform or less commonly subulate, (6.7–)8.1–11.4(−14.7) × 2.8–4.2 μm, l/w 1.9–3.5(−4.8), 1.9–2.8 μm wide at the base (n = 40). Conidia green, smooth, globose, subglobose or ellipsoid, 3.3–4.4(−5.0) × 3.2–4.2 μm, l/w 1.0–1.4 (n = 40). Chlamydospores rare. No distinct odour, no diffusing pigment observed.

On SNA after 72 h colony radius 31–35 mm, mycelium covering the plate after 6 d at 25 °C. Colony hyaline, radial, mycelium loose, aerial hyphae inconspicuous. Conidiation noted in pustules after 15 d, pustules not common, appearing around the original inoculum, loose, irregular in shape, white, turning green after 14 d. Chlamydospores rare. No distinct odour, no diffusing pigment observed.

*Additional strain examined*: CHINA, TIBET: Linzhi, Motuo County, elev. 1150 m, isolated from soil, September 2016, *K. Chen*, *TC968* (HMAS 248872).

*Notes*: *Trichoderma brevicrassum* grows fast on CMD and PDA (about 70 mm after 3 d at 25 °C) and has relatively short and wide phialides, which resemble *T. breve* and *T. crassum*. However, in comparison with the new species, *T. breve* produces less chlamydospores on PDA and smaller conidia (2.5–3.5 × 2.5–3.1 μm). *Trichoderma crassum* forms conidiophores with sterile elongations, shorter phialides (4.4–9.5 × 3.0–4.2 μm) and smaller conidia (3.7–5.3 × 2.6–3.7 μm)^22^. Phylogenetically, *T. brevicrassum* is closely related to *T. surrotundum* (Fig. 1), but the latter species has larger conidia (4.5–5.0 × 3.7–4.0 μm) and grows much slower on PDA at 25 °C (26–32 mm after 3 d)^7^.

**Figure 7:**
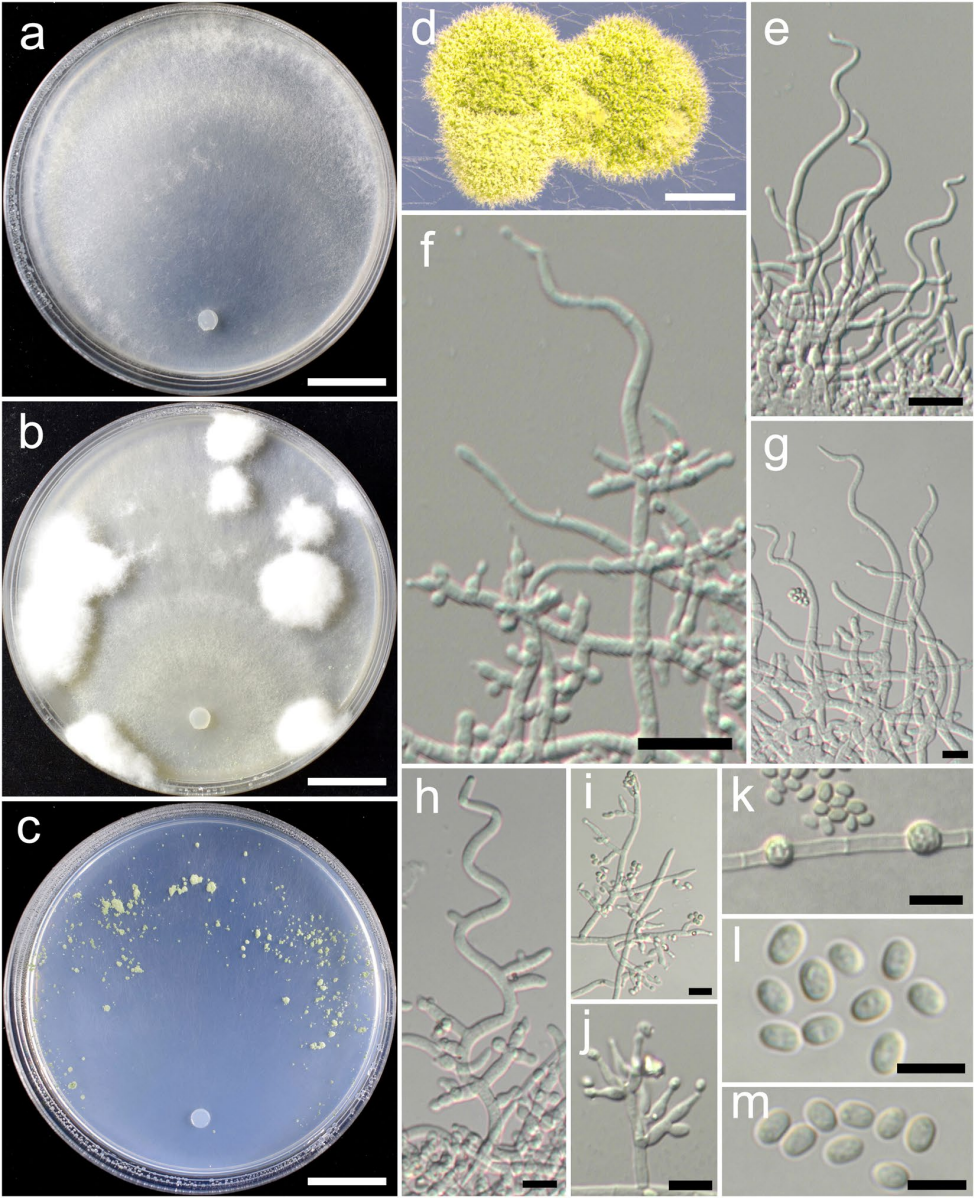
*Trichoderma byssinum* (HMAS 248838). (**a**–**c**) Cultures at 25 °C, 10 d (a. on CMD; b. on PDA; c. on SNA); (**d**) Conidial pustules (SNA, 7 d); (**e**–**h**) Pachybasium-like conidiophores and phialides (SNA, 7 d); (**i**,**j**) Verticillium-like synanamorph (PDA, 7 d); (**k**) Chlamydospores (SNA, 7 d); (**l**–**m**) Conidia (SNA, 7 d). Bars: a–c = 20 mm. d = 400 μm. e–f = 20 μm. g–k = 10 μm. l–m = 5 μm.

**Trichoderma byssinum** K. Chen & W.Y. Zhuang, **sp. nov**. Figure 7

Fungal Names: FN570403

*Etymology*: The specific epithet refers to the cotton-like aerial hyphae on PDA.

*Holotype*: CHINA, GUANGDONG: Zhaoqing, Fengkai County, elev. 292 m, isolated from soil, December 2014, *K. Chen*, *TC554* (HMAS 248838). Ex-type culture CGMCC 3.18393.

On CMD after 72 h colony radius 39–41 mm, mycelium covering the plate after 5 d at 25 °C. Colony hyaline, radial, indistinct zonate, aerial hyphae inconspicuous. Conidiation staring after 7 d, formed on aerial hyphae, rare. No chlamydospores observed. No distinct odour, no diffusing pigment observed.

On PDA after 72 h colony radius 35–37 mm, mycelium covering the plate after 6 d at 25 °C. Colony radial, indistinct zonate, aerial hyphae common, cotton-like aerial hyphae formed around the periphery of the colony, loose, up to 25 mm diam. Conidiation noted on aerial hyphae after 3 d, spreading in several concentric rings. Verticillium-like synanamorph found on aerial hyphae, short and simple. Phialides narrowly lageniform, rarely ampulliform, (6.9–) 8.3–11.7(−13.8) × 2.4–3.5 μm, l/w2.2–5.1, 1.4–2.8 μm wide at the base (n = 30). No chlamydospores observed. No distinct odour, no diffusing pigment observed.

On SNA after 72 h colony radius 30–32 mm, mycelium covering the plate after 6 d at 25 °C. Colony hyaline, radial, mycelium loose, aerial hyphae nearly lacking. Conidiation formed in pustules after 6 d, pustules common, more abundant with distance from the original inoculum, hemispherical, compact, 1–4 mm diam, white, turning green after 8 d, with hairs protruding beyond the surface. Conidiophores pachybasium-like, with a main axis, up to 150 μm, elongations sinuous, sterile or fertile at the tips, side branch arising from the base, short, paired or unpaired. Phialides solitary or in pairs, ampulliform, 5.0–8.3(−10.6) × 3.1–3.9 μm, l/w1.4–2.7, 1.7–2.8 μm wide at the base (n = 30). Conidia green, smooth, ellipsoid, 3.1–3.6 × 2.1–2.5 μm, l/w 1.3–1.7 (n = 40). Chlamydospores common, intercalary or terminal, variable in shape, ellipsoide, globose or oblong, 4.1–6.9(−9.0) × 3.5–6.9 μm, l/w 1.0–2.4 (n = 30). No distinct odour, no diffusing pigment observed.

*Additional strain examined*: CHINA, GUANGDONG: Zhaoqing, Fengkai County, elev. elev. 292 m, isolated from soil, September 2016, *K. Chen*, *TC555* (HMAS 248839).

*Notes*: *Trichoderma byssinum* is distinctive by its cotton-like aerial hyphae on PDA, which is rarely seen in *Trichoderma*. Phylogenetically, *T. byssinum* is closely related to *T. helicum* and *T. undatipile*. Compared with the new species, no synanamorph is noticed in *T. helicum* and the species produces much shorter phialides (2.7–5.8 × 2.5–4.4 μm) and smaller conidia (2.5–3.5 × 1.7–2.5 μm)^23^. *Trichoderma undatipile* differs in much higher growth rates on all three media, verticillium-like conidiophores, larger and regularly globose conidia (2.8–4.7 × 2.5–3.6 μm).

**Figure 8:**
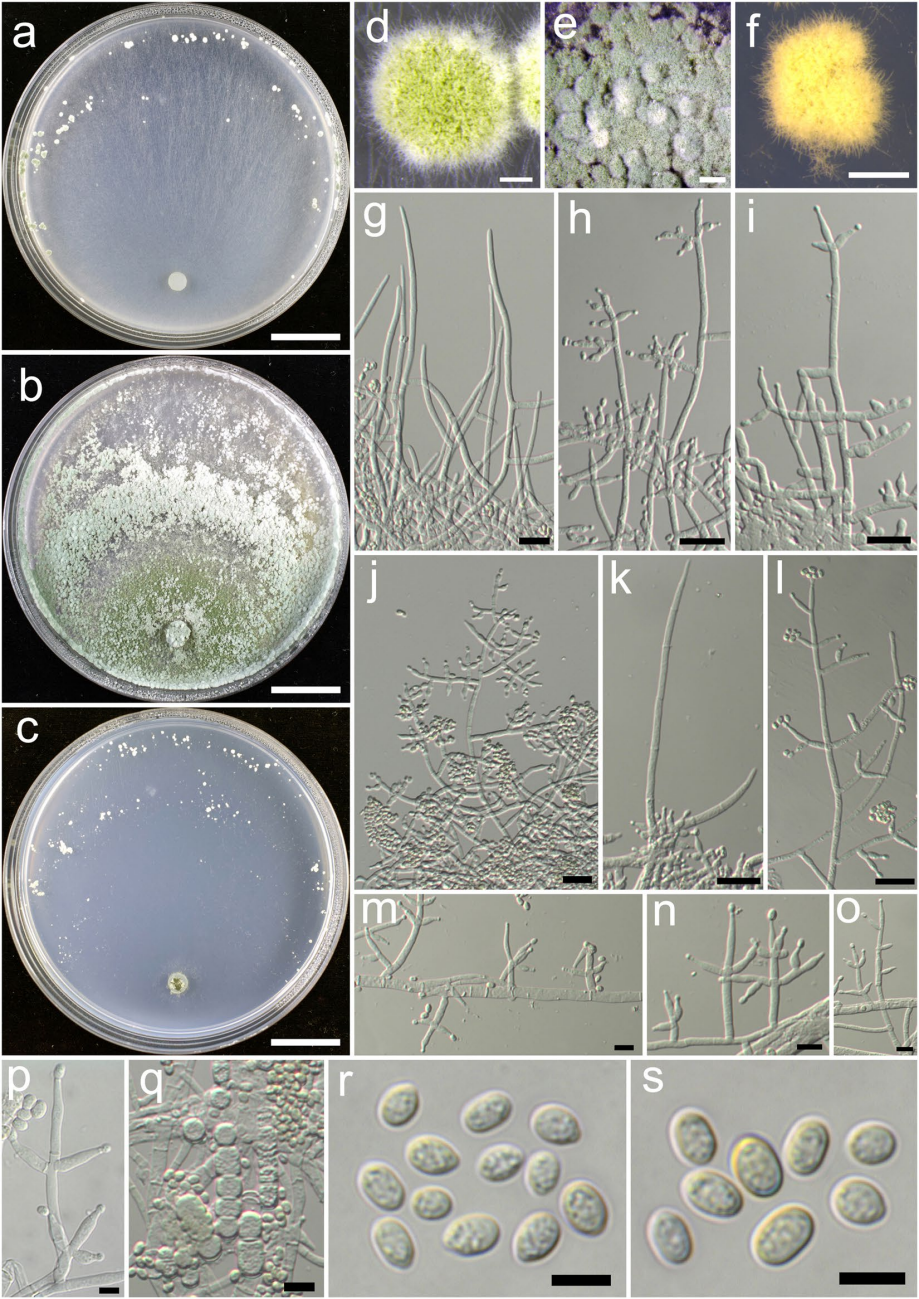
*Trichoderma chlamydosporicum* (HMAS 248850). (**a**–**c**) Cultures at 25 °C, 7 d (a. on CMD; b. on PDA; c. on SNA); (**d**–**f**) Conidial pustules (d. CMD, 7 d; e. PDA, 7 d; f. SNA, 7 d); (**g**–**k**) pachybasium-like conidiophores and phialides (PDA, 6 d); (**l**–**p**) Verticillium-like synanamorph (PDA, 5 d); (**q**) Chlamydospores (PDA, 5 d); (**r**,**s**) Conidia (PDA, 5 d). Bars: a–c = 20 mm. d = 300 μm. e = 800 μm. f = 400 μm. g–l = 20 μm. m–q = 10 μm. r–s = 5 μm.

**Trichoderma chlamydosporicum** K. Chen & W.Y. Zhuang, **sp. nov**. Figure 8

Fungal Names: FN570402

*Etymology*: The specific epithet refers to the numerous chlamydospores produced on PDA.

*Holotype*: CHINA, HEILONGJIANG: Daxinganling, Huzhong National Nature Reserve, elev. 740 m, isolated from soil, September 2015, *K. Chen*, *TC794* (HMAS 248850). Ex-type culture CGMCC 3.18401.

On CMD after 72 h colony radius 55–58 mm, mycelium covering the plate after 4 d at 25 °C. Colony hyaline, radial, mycelium loose, aerial hyphae inconspicuous. Conidiation formed in pustules after 5 d, pustules appearing around the periphery of the colony, hemispherical, compact, 1–3 mm diam, first white, turning grayish green after 6 d, with hairs protruding beyond the surface. No chlamydospores observed. No distinct odour, no diffusing pigment observed.

On PDA after 72 h colony radius 55–56 mm, mycelium covering the plate after 4 d at 25 °C. Colony not finely zonate, mycelium dense, aerial hyphae common. Conidiation noted after 2 d, formed in pustules, pustules spreading abundant in 2–3 indistinct concentric zones, hemispherical, compact, with hairs extending beyond the surface, hairs straight, tips unbranched or infrequently branched, typically sterile, sometimes fertile. Conidiophores in pustules pachybasium-like, often with a main axis, tips sterile or fertile, side branches arising from the base, solitary, sometimes paired, not of rebranching once. Phialides short, ampulliform, less commonly lageniform, straight or hooked, (5.5–)6.9–10.3(−11.7) × 3.1–4.1 μm, l/w (1.6–)2.0–3.2(−4.3), 2.1–2.8 μm wide at the base (n = 30). Verticillium-like synanamorph was noted on aerial hyphae, conidiophores shot and simple, typically unbranched, loosely disposed on aerial hyphae. Phialides variable in shape and size, lageniform to narrowly lageniform, sometimes ampulliform, (6.1–)10.3–15.0(−23.1) × 2.8–3.6 μm, l/w (1.8–)3.1–6.0(−8.3), 1.7–2.8 μm wide at the base (n = 30). Conidia green, smooth, ellipsoid, sometimes oblong, (3.6–)4.2–5.0(−5.8) × 2.8–3.6 μm, l/w 1.2–1.6(−1.9) (n = 40). Chlamydospores numerous, intercalary, typically globose, sometimes ellipsoid or oblong, 6.2–10.3(−12.4) × 5.5–8.6 μm, l/w 1.0–1.5(−1.9) (n = 30). No distinct odour, no diffusing pigment observed.

On SNA after 72 h colony radius 35–36 mm, mycelium covering the plate after 5 d at 25 °C. Colony hyaline, mycelium loose, aerial hyphae nearly lacking. Conidiation starting after 3 d, first formed on short erect conidiophores near the original inoculum, conidial pustules noted after 6 d, appearing around the periphery of the colony, hemispherical, 0.5–1 mm diam, white, turning green after 7 d, with hairs protruding beyond the surface. Chlamydospores rare. No distinct odour, no diffusing pigment observed.

*Additional strain examined*: CHINA, HEILONGJIANG: Daxinganling, Huzhong National Nature Reserve, elev. 740 m, isolated from soil, September 2015, *K. Chen*, *TC795* (HMAS 248851).

*Notes*: *Trichoderma chlamydosporicum* is featured by its numerous chlamydospores produced on PDA. This species also forms numerous compact, hemispherical conidial pustules on PDA that resembles *T. hamatum*. But it differs obviously from *T. hamatum* in the elongated conidiophores, which are straight and frequently fertile at the tips^14^. Phylogenetically, *T. chlamydosporicum* is closely related to *T. tibetense*, but the latter species differs in colony characteristics, slow growth on PDA, trichoderma-like conidiophores and smaller conidia (3.3–5.6 × 2.5–3.3)^18^.

**Figure 9:**
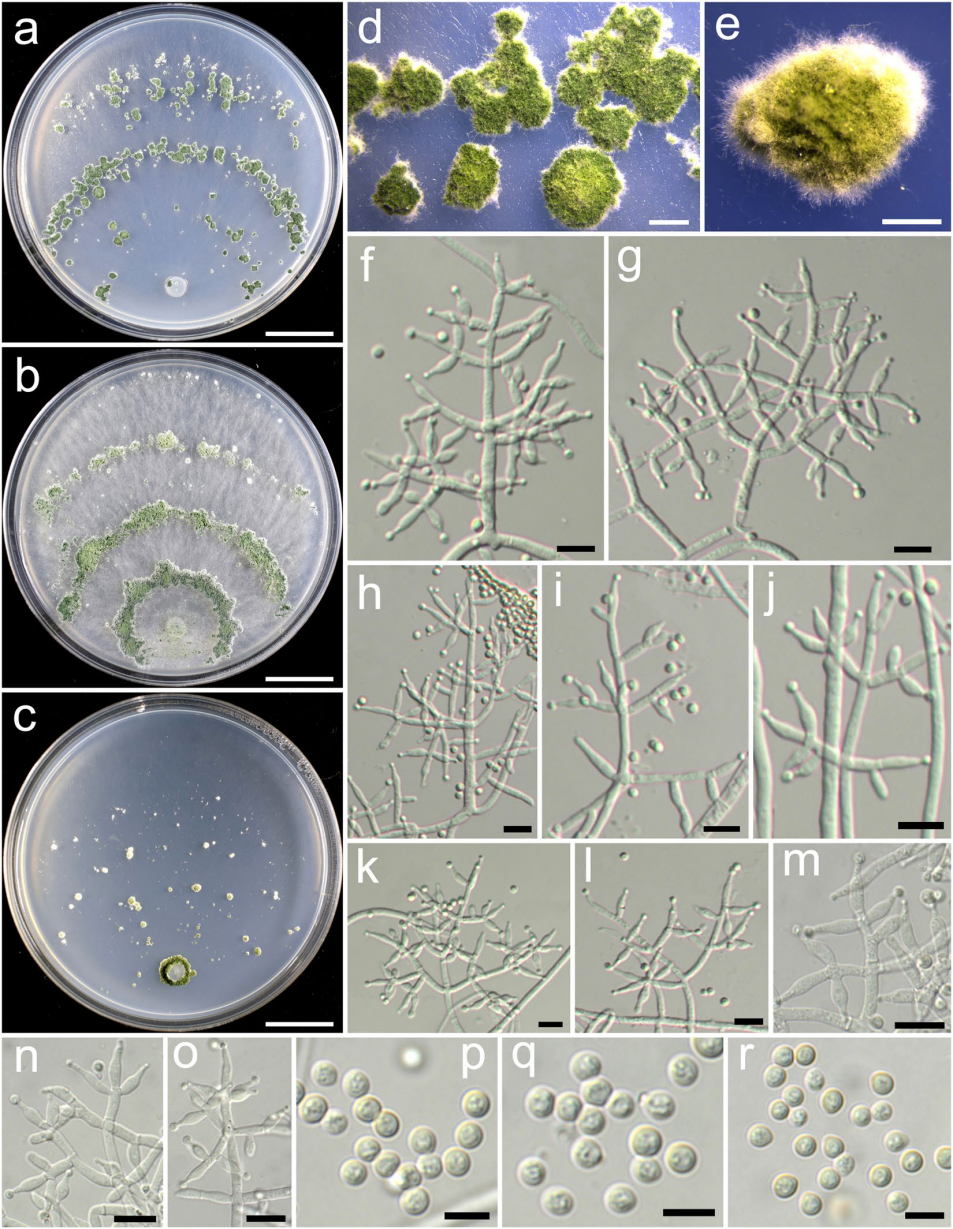
*Trichoderma concentricum* (HMAS 248833). (**a,c**) Cultures at 25 °C, 7 d (a. on CMD; b. on PDA; c. on SNA); (**d**,**e**) Conidial pustules (d. CMD, 7 d; e. SNA, 7 d); (**f**–**o**) Conidiophores and phialides (PDA, 6 d); (**p**–**r**) Conidia (p, q. PDA, 6 d; r. CMD, 6 d). Bars: a–c = 20 mm. d = 2 mm. e = 1.6 mm. f–o = 10 μm. p–r = 5 μm.

**Trichoderma concentricum** K. Chen & W.Y. Zhuang, **sp. nov**. Figure 9

Fungal Names FN570401

*Etymology*: The specific epithet refers to the concentric rings formed on CMD and PDA.

*Holotype*: CHINA, HUBEI: Shennongjia Natural Reserve, elev. 1775 m, isolated from soil, September 2014, *K. Chen*, *TC295* (HMAS 248833). Ex-type culture CGMCC 3.18389.

On CMD after 72 h colony radius 48–49 mm, mycelium covering the plate after 4 d at 25 °C. Colony hyaline, radial, circular, not finely zonate, aerial hyphae inconspicuous. Conidiation formed in pustules after 3 d, pustules abundant, spreading in 3–4 concentric rings, hemispherical to pulvinate, outline irregular, surface downy, 1–4 mm diam, white, turning green after 4 d. No chlamydospores observed. No distinct odour, no diffusing pigment observed.

On PDA after 72 h colony radius 39–42 mm, mycelium covering the plate after 5 d at 25 °C. Colony similar to CMD, but mycelium denser and aerial hyphae more common. Conidiation starting after 3 d, in aggregated pustules, pustules abundant, spreading in 3–4 concentric rings, aggregated, up to 5 mm in width of the concentric rings, with white droplets on the surface, first white, turning green after 4 d. Conidiophores symmetry, trichoderma-like, often with a main axis, side branches in acute or straight angles with main axis, typically paired, less commonly in whorls of 3, rebranching 1–3 times. Phialides formed solitary or paired, rarely in whorls of 3, lageniform to narrowly lageniform, 8.6–11.7(−13.6) × 2.5–3.6 μm, l/w 2.2–4.3(−5.4), 1.4–2.5 μm wide at the base (n = 40). Conidia green, smooth, with few small guttules, globose, sometimes subglobose, 2.6–3.3 × 2.6–3.3 μm, l/w 1.0–1.1(−1.2) (n = 40). No chlamydospores observed. No distinct odour, no diffusing pigment observed.

On SNA after 72 h colony radius 15–16 mm, mycelium covering the plate after 7 d at 25 °C. Colony hyaline, margin slightly lobed, not well defined, mycelium loose, aerial hyphae inconspicuous. Conidiation starting after 4 d, first formed on short erect conidiophores and aerial hyphae, pustules noted after 5 d, uniformly distributed throughout the colony, hemispherical, surface downy, 1–3 diam, white, turning green after 6 d. Chlamydospores rare. No distinct odour, no diffusing pigment observed.

*Additional strain examined*: CHINA, HUBEI: Shennongjia Natural Reserve, elev. 1775 m, isolated from soil, September 2016, *K. Chen*, *TC905* (HMAS 248858).

*Notes*: *Trichoderma concentricum* is characterized by the presence of distinct concentric pustules on CMD and PDA, globose conidia and lageniform phialides. Phylogenetically, *T. concentricum* is closely related to *T. corneum* and *T. ingratum*. Compared with the new species, *T. ingratum* differs in its colony morphology, unpleasant odour on PDA and slightly larger conidia (2.6–3.6 × 2.6–3.2 μm). *Trichoderma corneum* can be easily separated by its verticillium-like conidiophores, much longer phialides (8–24 × 1.5–3.0 μm) and ellipsoid conidia^24^.

**Figure 10:**
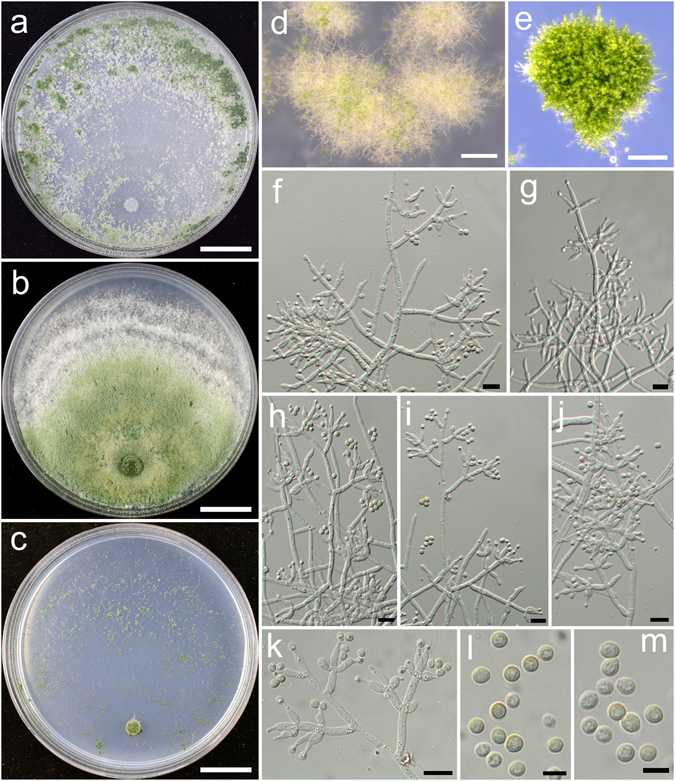
*Trichoderma ganodermatis* (HMAS 248856). (**a**–**c**) Cultures at 25 °C (a. on CMD, 10 d; b. on PDA, 7 d; c. on SNA, 10 d); (**d**,**e**) Conidial pustules (d. CMD, 7d; e. SNA, 7 d); (**f**–**k**) Conidiophores and phialides (PDA, 3 d); (**l**,**m**) Conidia (PDA, 3 d). Bars: a–c = 20 mm. d–e = 200 μm. f–k = 10 μm. l–m = 5 μm.

**Trichoderma ganodermatis** K. Chen & W.Y. Zhuang, **sp. nov**. Figure 10

Fungal Names: FN570400

*Etymology*: The specific epithet refers to the *Ganoderma* fruitbody from which the fungus was isolated.

*Holotype*: CHINA, HUNAN: Chenzhou, Mangshan National forest Park, elev. 1500 m, isolated from a *Ganoderma* on dead wood, October 2015, *X. C. Wang*, *TC877* (HMAS 248856). Ex-type culture CGMCC 3.18405.

On CMD after 72 h colony radius 24–34 mm, mycelium covering the plate after 5 d at 25 °C. Colony hyaline, radial, mycelium loose, aerial hyphae inconspicuous. Conidiation starting after 3 d, formed in pustules, pustules appearing around the periphery of the colony, hemispherical, loose, 0.5–2 mm diam, white, turning green after 5 d, with hairs protruding beyond the surface, straight, tips often branched and fertile. No chlamydospores observed. No distinct odour, no diffusing pigment observed.

On PDA after 72 h colony radius 22–27 mm, mycelium covering the plate after 6–7 d at 25 °C. Colony radial, not finely zonate, mycelium dense, aerial hyphae numerous, short, forming a dense downy mat throughout the colony. Conidiation noted after 2 d, formed on aerial hyphae, first near the original inoculum, spreading throughout the colony after 7 d. Conidiophores trichoderma-like, irregularly branched, often rebranching one time, main axis often ending in branched or unbranched elongations, tips sterile or fertile. Phialides typically formed in whorls of 3–4, not commonly solitary or paired, lageniform, sometimes subulate in terminal position on the axis, (5.8–)8.1–12.2(−15.0) × 2.2–3.9 μm, l/w (1.8–)2.4–4.4(−5.7), 1.7–3.1 μm wide at the base (n = 40). Conidia green, smooth, globose, subglobose or ellipsoid, (3.1–)3.9–4.4 × 3.1–3.5 μm, l/w 1.0–1.3 (n = 40). No chlamydospores observed. No distinct odour, no diffusing pigment observed.

On SNA after 72 h colony radius 12–20 mm, mycelium covering the plate after 8 d at 25 °C. Colony hyaline, indistinctly zonate, margin not well defined, mycelium loose, aerial hyphae inconspicuous. Conidiation starting after 3 d, effused on aerial hyphae and short erect conidiophores around the colony center, conidial pustules noted after 4 d, spreading uniformly throughout the colony, hemispherical, loose, 0.5–1 mm diam, white, turning green after 5 d. No chlamydospores observed. No distinct odour, no diffusing pigment observed.

*Additional strain examined*: CHINA, HUNAN: Chenzhou, Mangshan National forest Park, elev. 1500 m, isolated from a *Ganoderma* on dead wood, October 2015, *X. C. Wang*, *TC947* (HMAS 248869).

*Notes*: *Trichoderma ganodermatis* might be fungicolous since it was isolated from a fresh *Ganoderma* fruitbody. Several *Trichoderma* species have been reported as fungicolous, e.g. *T. hypoxylon*, *T. pleuroti*, *T. pleuroticola*, *T. songyi* and *T. stromaticum* ^25–28^. These species are either causing agents of green mold diseases in mushroom cultivation or mycoparasitic fungi on plant pathogens and have the potential in biocontrol. Phylogenetically, *T. ganodermatis* is closely related to *T. phyllostachydis* (Fig. 1). But the latter fungus grows faster on SNA at 25 °C (34–37 mm) and produces much shorter phialides (6.5–7.0 μm long) and conidia (2.3–3.0 μm long)^7^.

**Figure 11:**
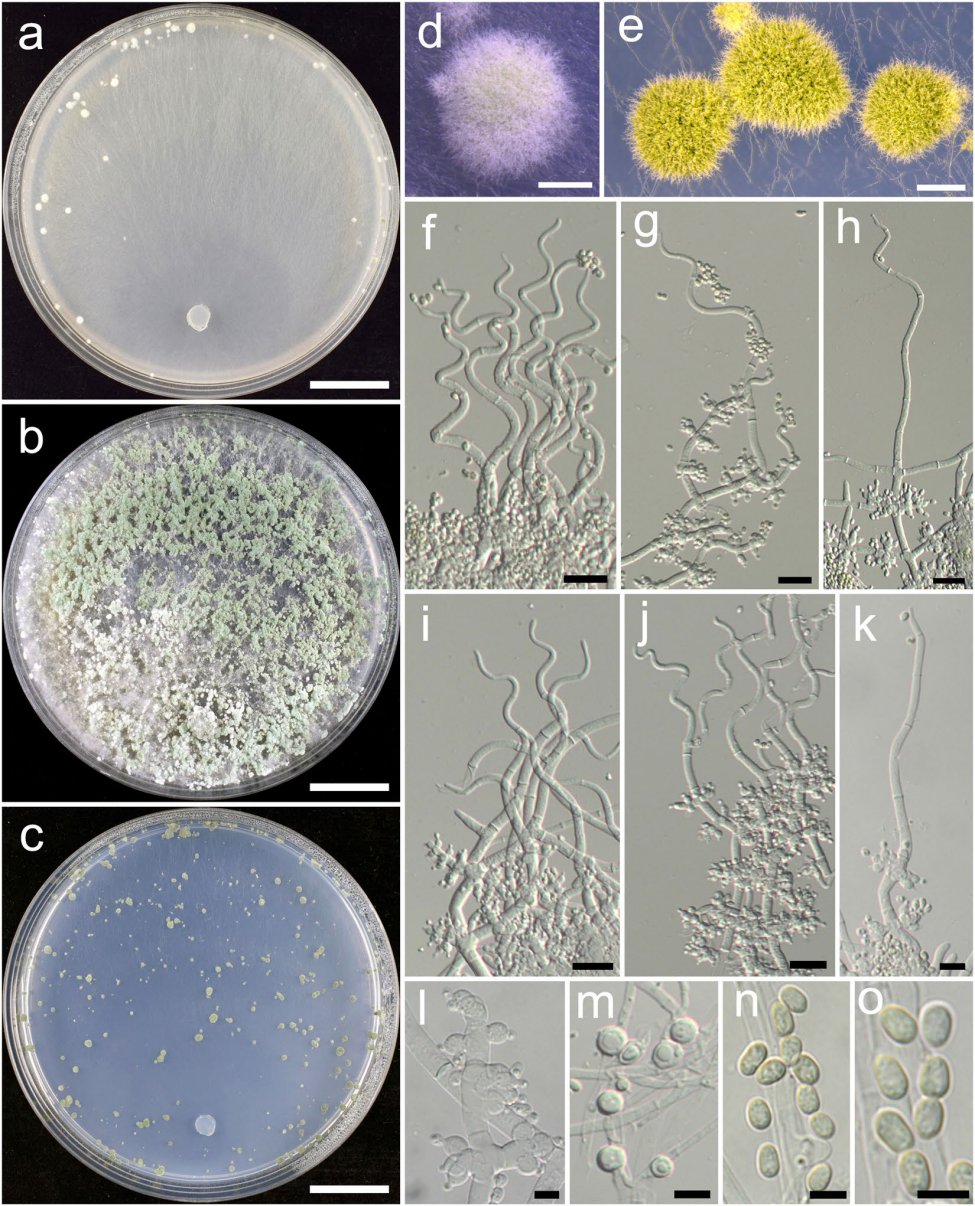
*Trichoderma hainanense* (HMAS 248837). (**a–c**) Cultures at 25 °C, 10 d (a. on CMD; b. on PDA; c. on SNA); (**d–e**) Conidial pustules (d. CMD, 7 d; e. SNA, 7 d); (**f–l**) Conidiophores and phialides (SNA, 6 d); (m) Chlamydospores (SNA, 6 d); (n–o) Conidia (SNA, 6 d). Bars: **a–c** = 20 mm.**d–e** = 400 μm. **f–j** = 20 μm. **k**, **m** = 10 μm. **l, n, o** = 5 μm.

**Trichoderma hainanense** K. Chen & W.Y. Zhuang, **sp. nov**. Figure 11

Fungal Names: FN570397

*Etymology*: The specific epithet refers to the type locality.

*Holotype*: CHINA, HAINAN: Jianfengling National Forest Park, elev. elev. 500 m, isolated from soil, December 2014, *K. Chen*, *TC468* (HMAS 248837). Ex-type culture CGMCC 3.18392.

On CMD after 72 h colony radius 58–62 mm, mycelium covering the plate after 4 d at 25 °C. Colony hyaline, radial, mycelium loose, aerial hyphae inconspicuous. Conidiation noted in pustules after 7 d, pustules appearing around the periphery of the colony, hemispherical, loose, remaining discrete, 1–3 mm diam, white, turning green after 10 d, with hairs protruding beyond the surface, hairs long, sinuous, tips unbranched, sterile or infrequently fertile. Chlamydospores not common. No distinct odour, no diffusing pigment observed.

On PDA after 72 h colony radius 68–70 mm, mycelium covering the plate after 3 d at 25 °C. Colony green, mycelium dense, aerial hyphae abundant. Conidiation starting after 8 d, formed in pustules, pustules abundant, uniformly distributed throughout the colony, hemispherical or pulvinate, compact, white, turning green after 9 d, with hairs extending beyond the surface. No chlamydospores observed. No distinct odour, no diffusing pigment observed.

On SNA after 72 h colony radius 39–46 mm, mycelium covering the plate after 5 d at 25 °C. Colony hyaline, radial, mycelium loose, aerial hyphae nearly lacking. Conidiation noted after 4 d in pustules, pustules common, spreading uniformly throughout the colony, hemispherical, compact, remaining discrete, 1–4 mm diam, first white, turning green after 5 d, with hairs protruding beyond the surface, hairs short, sinuous, tip often sterile, sometimes fertile with one phialides. Conidiophores pachybasium-like, with a distinct main axis, fertile or sterile elongations up to 200 μm, side braches arising from the base, short, paired or unpaired. Phialides densely disposed on side braches, paired or in whorls of 3–4, ampulliform, 5.3–9.7 × 3.6–4.7 μm, l/w1.3–2.3, 1.9–4.0 μm wide at the base (n = 40). Conidia green, smooth, ellipsoid, 3.9–5.0(−5.5) × 2.6–3.1 μm, l/w 1.4–1.8(−2.0) (n = 40). Chlamydospores numerous, intercalary or terminal, variable in shape and size, typically ellipsoid, sometimes globose or oblong, 4.8–10.3(−15.2) × 4.8–8.3 μm, l/w 1.0–1.5(−2.0) (n = 30). No distinct odour, no diffusing pigment observed.

*Additional strain examined*: CHINA, HAINAN: Jianfengling National Forest Park, elev. 500 m, isolated from soil, September 2016, *K. Chen*, *TC944* (HMAS 248866).

*Notes*: The conidiophores of *T. hainanense* are firmly aggregated in pustules. This species forms sinuous hairs beyond the surface of pustules, which is similar to species in the Stromaticum Clade, but they are phylogenetically distantly related and do not produce green ascospores^29^. Phylogenetically, *T. hainanense* is closely related to *T. silvae-virgineae*, but the latter fungus differs in very slow-growth at 25 °C (42–43 mm on CMD, 34–35 mm on PDA and 34–35 mm on SNA after 3 d) and formation of straight hairs beyond the surface of pustules^19^.

**Figure 12:**
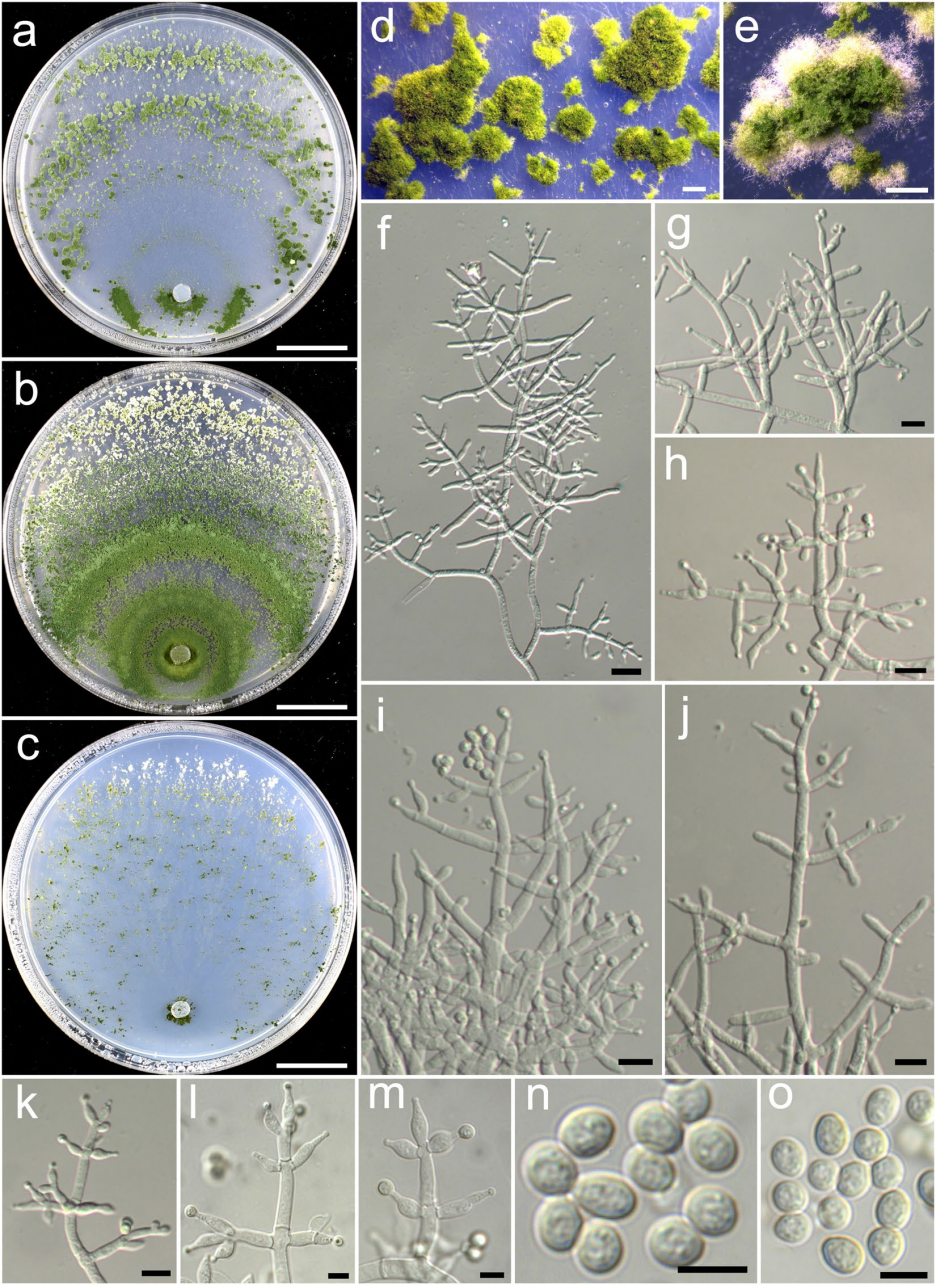
*Trichoderma hengshanicum* (HMAS 248852). (**a–c**) Cultures at 25 °C (a. on CMD, 7 d; b. on PDA, 7 d; c. on SNA, 12 d); (**d–e**) Conidial pustules (d. CMD, 7d; e. PDA, 7 d); (**f–m**) Conidiophores and phialides (PDA, 4 d); (**n–o**) Conidia (PDA, 4 d). Bars: a–c = 20 mm. d–e = 400 μm. f = 20 μm. g–k = 10 μm. l–o = 5 μm.

**Trichoderma hengshanicum** K. Chen & W.Y. Zhuang, **sp. nov**. Figure 12

Fungal Names FN570398

*Etymology*: the specific epithet refers to the type locality.

*Holotype*: CHINA, HUNAN: Hengyang, Hengshan National Nature Reserve, elev. 600 m, isolated from soil, November 2015, *K. Chen*, *TC842* (HMAS 248852). Ex-type culture CGMCC 3.18402.

On CMD after 72 h colony radius 53–56 mm, mycelium covering the plate after 4 d at 25 °C. Colony hyaline, radial, not finely zonate, mycelium loose, aerial hyphae common. Conidiation starting after 2 d, formed in pustules, pustules numerous, spreading in 3–4 concentric zones, irregular in shape, loose, outline not well defined, aggregated near the original inoculum and remaining discrete in distant areas, 1–4 mm diam, green. No chlamydospores observed. No distinct odour, no diffusing pigment observed.

On PDA after 72 h colony radius 41–47 mm, mycelium covering the plate after 7 d at 25 °C. Colony green, zonate, mycelium loose, aerial hyphae abundant, more abundant at the colony center. Conidiation starting after 2 d, formed on aerial hyphae near the colony center or in conidial pustules around the periphery of the colony, pustules irregular in shape, outline not well defined, 1–3 mm diam, green. Conidiophores trichoderma-like, often asymmetry, branches solitary, paired or in whorls of 3. Phialides formed solitary, paired or in whorl, variable in shape, lageniform, sometimes ampulliform or subulate, 7.2–12.8(−15.8) × 2.8–4.0 μm, l/w 1.9–4.8, 1.7–2.8 μm wide at the base (n = 40). Conidia green, smooth, ellipsoid, globose or subglobose, 3.3–4.4(−6.1) × 3.2–3.8(−4.2) μm, l/w 1.0–1.5 (n = 40). No chlamydospores observed. No distinct odour, no diffusing pigment observed.

On SNA after 72 h colony radius 40–42 mm, mycelium covering the plate after 5 d at 25 °C. Colony hyaline, radial, inconspicuous zonate, mycelium loose, aerial hyphae common. Conidiation starting after 2 d, formed on short erect conidiophores and aerial hyphae, small granules noted after 4 d, formed on aerial hyphae, spreading in inconspicuous concentric rings, 0.5–1 mm diam, green. No chlamydospores observed. No distinct odour, no diffusing pigment observed.

*Additional strain examined*: CHINA, HUNAN: Hengyang, Hengshan National Nature Reserve, elev. 600 m, isolated from soil, November 2015, *K. Chen*, *TC843* (HMAS 248853).

*Notes*: Phylogenetically, *T. hengshanicum* forms a sister group with *T. aggressivum*, *T. epimyces*, *T. priscilae* and *T. rufobrunneum* (Fig. 1). In comparison of the four related species, *T. aggressivum* differs in smaller conidia (3.2–3.3 × 2.8–2.9 μm); *T. epimyces* grows slower than the new species in addition to producing smaller conidia (3.0–3.7 × 2.7–3.0 μm); *T. priscilae* can be easily separated by much shorter phialides (5.5–8.2 μm) and *T. rufobrunneum* by higher growth rate on SNA (21–25 mm at 25 °C)^12, 14, 21, 30^.

**Figure 13:**
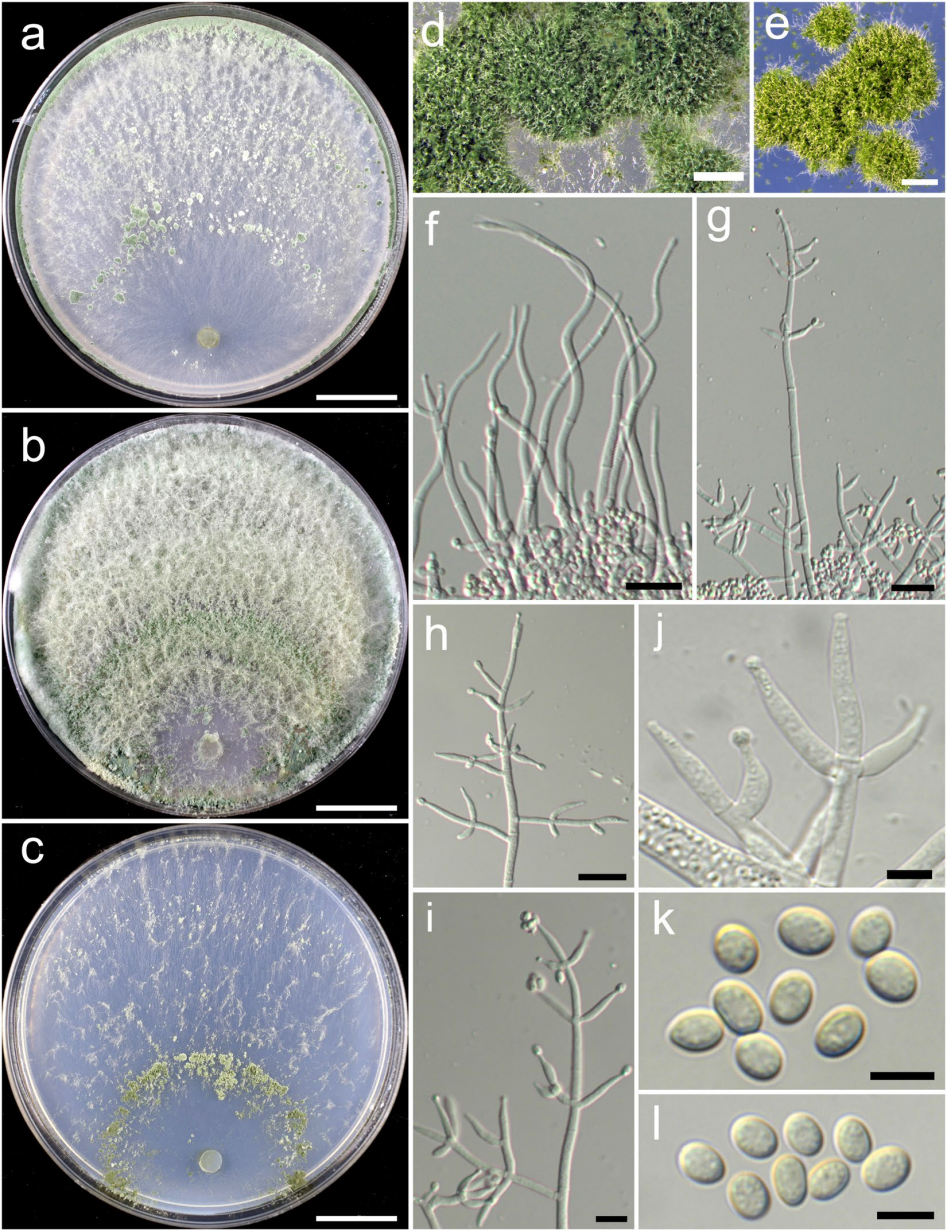
*Trichoderma hirsutum* (HMAS 248834). (**a–c**) Cultures at 25 °C, 7 d (a. on CMD; b. on PDA; c. on SNA); (**d–e**) Conidial pustules (d. CMD, 7 d; e. SNA, 7 d); (**f–j**) Conidiophores and phialides (PDA, 4 d); (k–l) Conidia (PDA, 4 d). Bars: a–c = 20 mm. d–e = 400 μm. f–h = 20 μm. i = 10 μm. j–l = 5 μm.

**Trichoderma hirsutum** K. Chen & W.Y. Zhuang, **sp. nov**. Figure 13

Fungal Names: FN570399

*Etymology*: The specific epithet refers to the hairy colony on PDA.

*Holotype*: CHINA, HUBEI: Shennongjia Natural Reserve, elev. 1200 m, isolated from soil, September 2014, *K. Chen*, *TC334* (HMAS 248834). Ex-type culture CGMCC 3.18390.

On CMD after 72 h colony radius 41–45 mm, mycelium covering the plate after 4 d at 25 °C. Colony hyaline, radial, not zonate, mycelium dense, aerial hyphae common, absent at colony center. Conidiation starting after 3 d, formed on aerial hyphae and in pustules. Pustules forming in 2–3 concentric rings, aggregated at colony margin and discrete around the original inoculum, hemispherical, compact, 1–3 mm diam, green. Hairs protruding beyond the surface of the pustules, sinuous, tip often unbranched, sterile or fertile. Conidiophores in pustules pachybasium-like, comprising a long main axis, often produce phialides from the tips, fertile branches arising from the base of hairs, paired or solitary. Conidiophores in aerial hyphae verticillium-like, short and simple. Phialides long and thin, narrowly lageniform, rarely subulate, (8.6–)11.4–18.3(−23.5) × 2.5–3.1 μm, l/w 3.2–6.3(−9.7), 1.9–3.3 μm wide at the base (n = 40). Conidia green, smooth, ellipsoid, 3.9–4.7(−5.4) × 2.9–3.9 μm, l/w 1.2–1.4(−1.6) (n = 40). No chlamydospores observed. No distinct odour, no diffusing pigment observed.

On PDA after 72 h colony radius 43–45 mm, mycelium covering the plate after 4 d at 25 °C. Colony circular, not finely zonate, mycelium dense. Aerial hyphae numerous, long and wooly, forming a loose, floccose, zonate mat, absent near the original inoculum. Conidiation starting after 3 d, numerous, effuse in aerial hyphae. No chlamydospores observed. No distinct odour, no diffusing pigment observed.

On SNA after 72 h colony radius 34–37 mm, mycelium covering the plate after 5 d at 25 °C. Colony similar to CMD, but with less aerial hyphae. Conidiation starting after 3 d, first formed on aerial hyphae, conidial pustules noted after 4 d, spreading in concentric rings around the original inoculum, hemispherical to spherical, compact, 0.5–2 mm diam, green. Hairs extending beyond the surface of the pustules, sinuous or spiraled, tip often unbranched, sterile or fertile. No chlamydospores observed. No distinct odour, no diffusing pigment observed.

*Additional strain examined*: CHINA, HUBEI: Shennongjia Natural Reserve, elev. 1200 m, isolated from soil, September 2016, *K. Chen*, *TC906* (HMAS 248859).

*Notes*: *Trichoderma hirsutum* is characterized by compact pustules with hairs on the surface and relatively large conidia. Phylogenetically, it is closely related to *T. catoptron* and *T. pseudogelatinosum*. However, *T. catoptron* produces much shorter phialides (5.5–7.2 × 3.2–4.2 μm) and much complicated conidiophore branches^7^. *Trichoderma pseudogelatinosum* differs in much slower growth (17.3–25.2 mm on PDA and 21.1–22.6 mm on SNA after 3 d at 25 °C), gliocladium- to verticillium-like conidiophores and much shorter phialides (6.7–10.1 × 2.2–2.7 μm)^31^.

**Figure 14:**
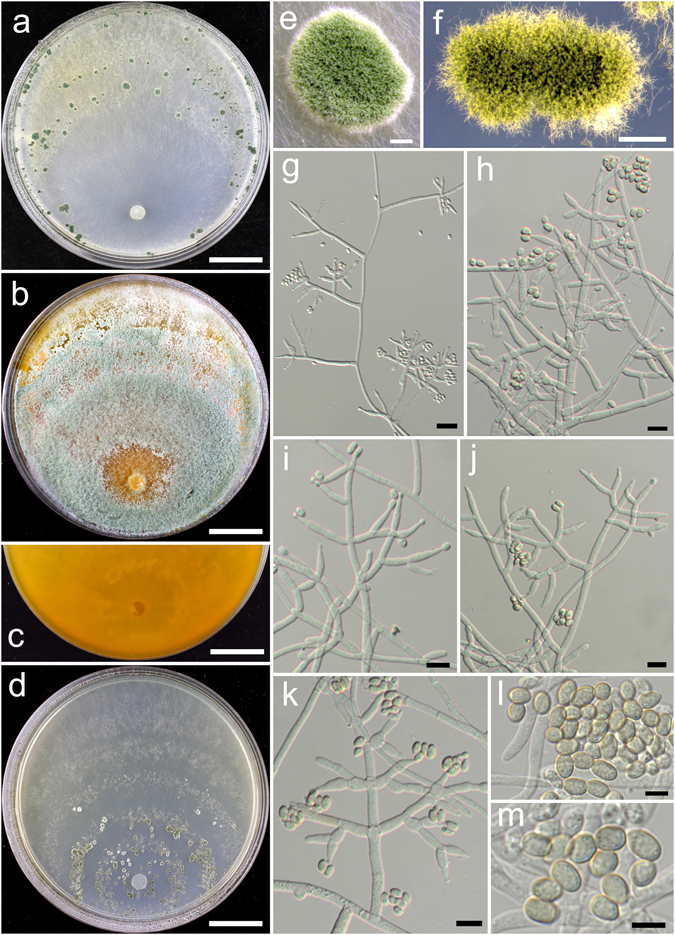
*Trichoderma hunanense* (HMAS 248841). (**a**–**d**) Cultures at 25 °C, 7 d (a. on CMD; b. on PDA; c. on PDA, reverse; d. on SNA); (**e**,**f**) Conidial pustules (e. CMD, 7 d; f. SNA, 7 d); (**g**–**k**) Conidiophores and phialides (PDA, 4 d); (**l**–**m**) Conidia (CMD, 4 d). Bars: a–d = 20 mm. e = 600 μm. f = 800 μm. g = 20 μm. h–k = 10 μm. l–m = 5 μm.

**Trichoderma hunanense** K. Chen & W.Y. Zhuang, **sp. nov**. Figure 14

Fungal Names: FN570395

*Etymology*: The specific epithet refers to the type locality.

*Holotype*: CHINA, HUNAN: Zhangjiajie, Badagongshan National Nature Reserve, elev. 1400 m, isolated from soil, January 2015, *K. Chen*, *TC579* (HMAS 248841). Ex-type culture CGMCC 3.18395.

On CMD after 72 h colony radius 48–49 mm, mycelium covering the plate after 4 d at 25 °C. Colony hyaline, radial, not finely zonate, aerial hyphae common, appearing in several concentric rings, denser at the colony margin, absent in colony center. Conidiation starting after 5 d, formed on aerial hyphae and in pustules, pustules spreading abundant around the periphery of the colony, pulvinate, 1–4 mm diam, first white, turning green after 8 d, with hairs protruding beyond the surface, hairs short, straight, infrequently branched at the tips. No chlamydospores, no distinct odour observed. Light yellowish pigment noted at the periphery of the colony.

On PDA after 72 h colony radius 46–47 mm, mycelium covering the plate after 5 d at 25 °C. Colony radial, indistinct zonate, aerial hyphae spreading abundant throughout the colony. Conidiation starting after 3 d, formed numerous on aerial hyphae, blue- green. Conidiophores trichoderma-like to veritcillium-like, short and simple, rebranching 1–2 times. Phialides loosely disposed, solitary or paired, lageniform to narrowly lageniform, sometimes subulate, (8.3–)11.1–15.3(−21.4) × 3.1–3.9 μm, l/w 2.1–5.5, 2.2–3.3 μm wide at the base (n = 40). Conidia green, smooth, ellipsoid, less commonly oblong or globose, (3.6–)4.2–5.6 (−7.5) × 3.1–3.9 μm, l/w (1.0–)1.2–1.6(−2.1) (n = 40). No chlamydospores, no distinct odour observed. Deep yellow odour noted.

On SNA after 72 h colony radius 27–28 mm, mycelium covering the plate after 7 d at 25 °C. Colony hyaline, radial, finely zonate, aerial hyphae appearing in 6–7 concentric rings. Conidiation starting after 3 d, first on aerial hyphae around the original inoculum, conidial pustules noted after 4 d, spreading in concentric rings, pulvinate, 1–3 mm diam, white, turning green after 5 d, with hairs extending beyond the surface, hairs short, straight, infrequently branched at the tips. No chlamydospores observed. No distinct odour observed. Light yellowish pigment noted.

*Additional strain examined*: CHINA, HUNAN: Badagongshan National Nature Reserve, elev. 1400 m, isolated from soil, September 2016, *K. Chen*, *TC945* (HMAS 248867).

#### *Notes:*

*Trichoderma hunanense* is recognizable by the deep yellow pigment produced in PDA (colony reverse view), which resembles *T. fertile*. But *T. hunanese* can be readily distinguished by slender and narrowly lageniform phialides instead of ampulliform in *T. fertile* ^22^. Phylogenetically, *T. hunanense* is closely related to *T. spirale* and *T. longisporum*. In comparison with the new species, *T. spirale* produces pachybasium-like conidiophores with distinct elongations, shorter phialides (3.3–5.2 × 2.8–4.0 μm) and smaller conidia (3.0–4.4 × 1.8–2.7 μm)^22^. *Trichoderma longisporum* differs in pachybasium-like conidiophores, ampulliform phialides and much longer conidia (5.0–6.4 × 2.6–3.1 μm).

**Figure 15:**
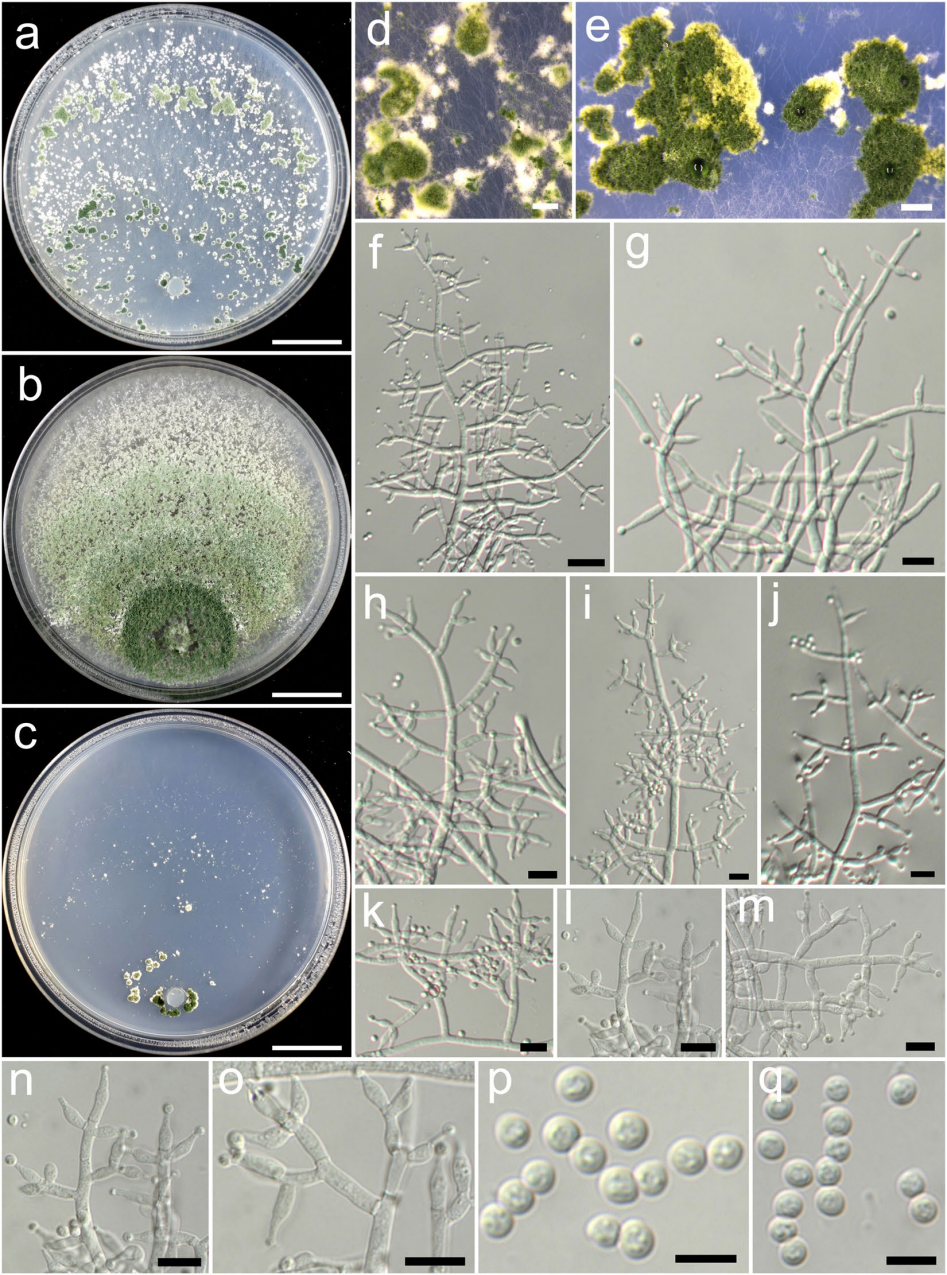
*Trichoderma ingratum* (HMAS 248822). (**a**–**c**) Cultures at 25 °C, 7 d (a. on CMD; b. on PDA; c. on SNA); (**d**,**e**) Conidial pustules (d. CMD, 7 d; e. SNA, 7 d); (**f**–**o**) Conidiophores and phialides (PDA, 4 d); (**p**–**q**) Conidia (PDA, 4 d). Bars: a–c = 20 mm. d–e = 500 μm. f = 20 μm. g–o = 10 μm. p–q = 5 μm.

**Trichoderma ingratum** K. Chen & W.Y. Zhuang, **sp. nov**. Figure 15

Fungal Names FN570396

*Etymology*: The specific epithet refers to the unpleasant odour on CMD.

*Holotype*: CHINA, SICHUAN: Ngawa Tibetan and Qiang Autonomous Prefecture, Rangtang County, elev. 3100 m, isolated from soil, August 2014, *K. Chen*, *TC34* (HMAS 248822). Ex-type culture CGMCC 3.18386.

On CMD after 72 h colony radius 52–54 mm, mycelium covering the plate after 4 d at 25 °C. Colony hyaline, radial, not zonate, aerial hyphae common. Conidiation starting after 3 d, formed on aerial hyphae and in pustules, pustules abundant, spreading throughout the colony, pulvinate with irregular outline, up to 6 mm diam, margin white, center green. No chlamydospores observed. Odour unpleasant, no diffusing pigment observed.

On PDA after 72 h colony radius 51–54 mm, mycelium covering the plate after 4 d at 25 °C. Colony circular, not finely zonate, mycelium dense. Aerial hyphae abundant, forming a dense, zonate, floccose mat, denser in colony center. Conidiation starting after 3 d, effuse in aerial hyphae or in loosely deposed granules, more abundant along the concentric rings. Conidiophores symmetry, trichoderma-like, often with a distinct main axis up to 160 μm, branches mostly unpaired, arising in right-angles or inclined upwards with the axis, not or rebranching once. Phialides lageniform, rarely ampliform or subulate, (6.9–)8.3–12.4(−15.9) × 2.8–3.5(−3.9) μm, l/w 2.5–4.5(−5.8), 1.7–2.8 μm wide at the base (n = 40). Conidia light green, smooth, mostly globose, 2.6–3.6 × 2.6–3.2 μm, l/w 1.0–1.1(−1.2) (n = 40). No chlamydospores observed. Odour strongly coconut-like, no diffusing pigment observed.

On SNA after 72 h colony radius 32–37 mm, mycelium covering the plate after 7 d at 25 °C. Colony similar to CMD, but aerial hyphae not common. Conidiation starting after 3 d, formed on aerial hyphae and in pustules, pustules formed in inconspicuous broad concentric rings around the original inoculum, denser at the colony center, hemispherical to pulvinate, 1–3 mm diam, first white, turning grayish green after 4 d, with green droplets on the surface. No chlamydospores observed. No distinct odour, no diffusing pigment observed.

*Additional strains examined*: CHINA, HUBEI: Shennongjia Natural Reserve, elev. 2280 m, isolated from soil, September 2014, *K. Chen*, *TC183* (HMAS 248824), *TC212* (HMAS 248826), *TC217* (HMAS 248827); CHINA, TIBET: Linzhi, Lulang, elev. 3340 m, isolated from soil, September 2016, *K. Chen*, *TC980* (HMAS 248873).

*Notes*: *Trichoderma ingratum* inhabits in high attitude forests of central and southwestern China, which is similar to *T. alpinum*. But *T. alpinum* differs in shorter phialides and larger conidia, and do not produce unpleasant odour in culture. Phylogenetically, *T. ingratum* is closely related to *T. corneum*, but the latter species can be easily separated by verticillium-like conidiophores and longer phialides (8–24 × 1.5–3.0 μm)^24^.

**Figure 16:**
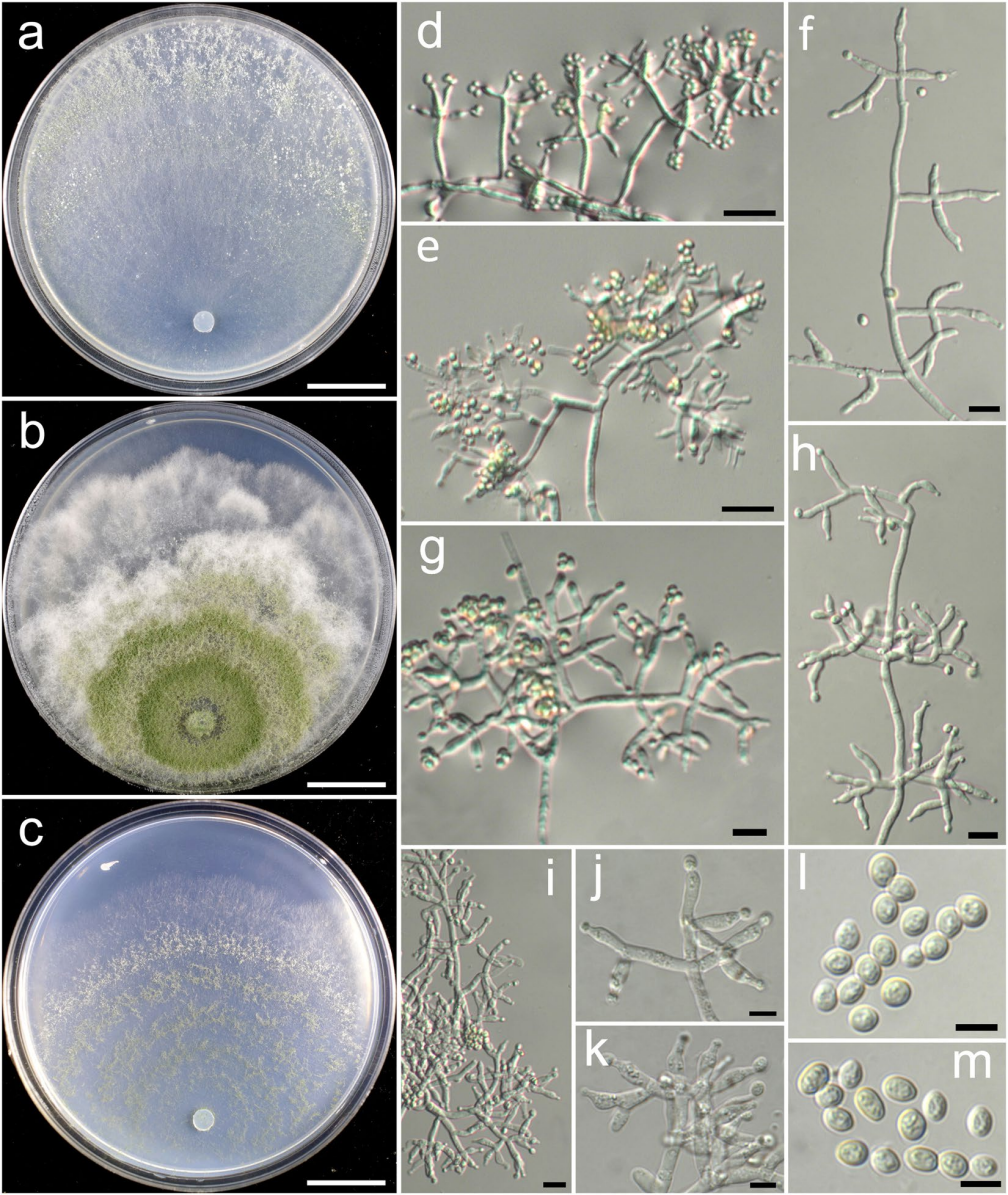
*Trichoderma liberatum* (HMAS 248831). (**a**–**c**) Cultures at 25 °C, 7 d (a. on CMD; b. on PDA; c. on SNA); (**d**–**k**) Conidiophores and phialides (PDA, 5 d); (**l**,**m**) Conidia (PDA, 5 d). Bars: a–c = 20 mm. d–e = 20 μm. f–i = 10 μm. j–m = 5 μm.

**Trichoderma liberatum** K. Chen & W.Y. Zhuang, **sp. nov**. Figure 16

Fungal Names FN570394

*Etymology*: The specific epithet refers to the loosely disposed branches of conidiophores.

*Holotype*: CHINA, HUBEI: Shennongjia Natural Reserve, elev. 2800 m, isolated from soil, September 2014, *K. Chen*, *TC253* (HMAS 248831). Ex-type culture CGMCC 3.18388.

On CMD after 72 h colony radius 37–38 mm, mycelium covering the plate after 5 d at 25 °C. Colony hyaline, radial, circular, mycelium dense, aerial hyphae common, more abundant with distance from the original inoculum. Conidiation starting after 3 d, effused on aerial hyphae or in small granules, denser along the colony margin. No chlamydospores observed. No distinct odour, no diffusing pigment observed.

On PDA after 72 h colony radius 31–33 mm, mycelium covering the plate after 7 d at 25 °C. Colony dense, not finely zonate, circular, margin not well defined and slightly wavy. Aerial hyphae numerous, forming a loose floccose mat, denser in the colony center. Conidiation starting after 2 d, on aerial hyphae around the original inoculum, numerous, white, turning green after 3 d. Conidiophores simple, asymmetry, verticillium-like, often comprising a main axis, side branches loosely disposed, paired or unpaired, in right or acute angles with the main axis, not or rebranching one time. Phialides solitary, less commonly paired or in whorls of 3, narrowly lageniform, often hooked, 8.3–13.9 × 2.8–3.9(−4.5) μm, l/w 2.4–4.3(−5.0), 1.9–2.5 μm wide at the base (n = 40). Conidia green, smooth, with several minute guttules, variable in shape, ellipsoid, less commonly globose, oval or oblong, (2.8–)3.5–4.7(−6.7) × 2.8–3.6 μm, l/w (1.0–)1.2–1.5(−2.2) (n = 40). No chlamydospores observed. No distinct odour, no diffusing pigment observed.

On SNA after 72 h colony radius 15–21 mm, mycelium covering the plate after 10 d at 25 °C. Colony hyaline, zonate, mycelium loose, margin not well defined. Aerial hyphae common, denser along the concentric rings. Conidiation starting after 3 d, effuse in aerial hyphae, spreading in 4–5 concentric zones. No chlamydospores observed. No distinct odour, no diffusing pigment observed.

*Additional strain examined*: CHINA, HUBEI: Shennongjia Natural Reserve, elev. 2800 m, isolated from soil, September 2014, *K. Chen*, *TC254* (HMAS 248832).

*Notes*: *Trichoderma liberatum* well-located in the Harzianum Clade forms verticillium-like conidiophores with the side branches loosely disposed. The conidiophore branching pattern is somewhat similar to *T. pararogersonii* in the Viride Clade. But the latter fungus produces hyaline ascospores and is remotely related. Phylogenetically, *T. liberatum* is associated with *T. pseudodensum* and *T. zayuense*, but *T. pseudodensum* can be easily separated by its densely disposed conidiophore branches, while *T. zayuense* differs in its slender and longer phialides (11.1–15.3 μm).

**Figure 17:**
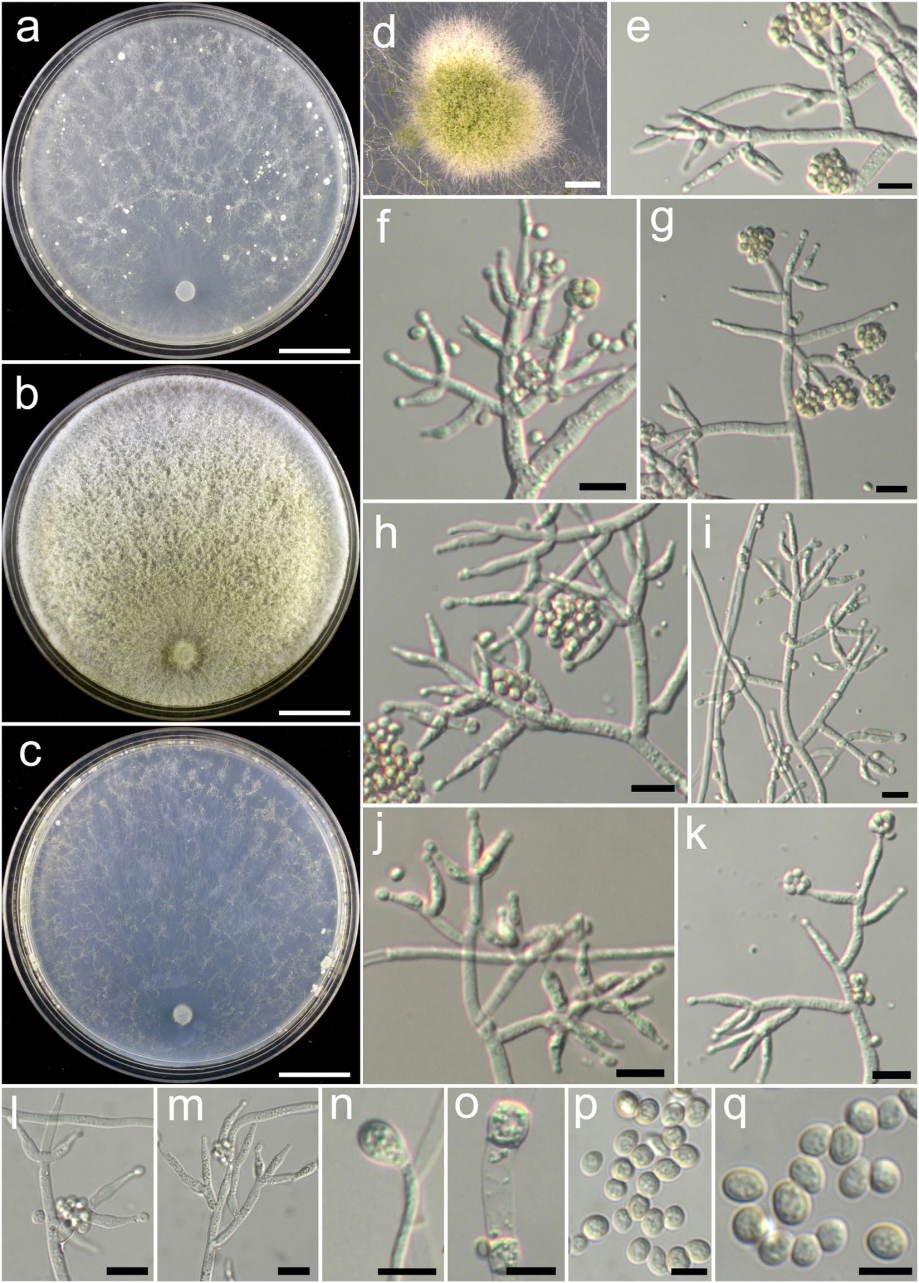
*Trichoderma linzhiense* (HMAS 248846). (**a**–**c**) Cultures at 25 °C, 7 d (a. on CMD; b. on PDA; c. on SNA); (**d**) Conidial pustules (CMD, 7 d); (**e**–**m**) Conidiophores and phialides (PDA, 5 d); (**n**–**o**) Chlamydospores (CMD, 5 d); (**p**–**q**) Conidia (PDA, 5 d). Bars: a–c = 20 mm. d = 400 μm. e–o = 10 μm. p–q = 5 μm.

**Trichoderma linzhiense** K. Chen & W.Y. Zhuang, **sp. nov**. Figure 17

Fungal Names FN570392

*Etymology*: The specific epithet refers to the type locality.

*Holotype*: CHINA, TIBET: Linzhi, Lulang, elev. 3250 m, isolated from soil, August 2015, *K. Chen*, *TC742* (HMAS 248846). Ex-type culture CGMCC 3.18399.

On CMD after 72 h colony radius 51–60 mm, mycelium covering the plate after 4 d at 25 °C. Colony hyaline, not zonate, mycelium loose, aerial hyphae common, wooly, long, extending to the Petri-dish cover. Conidiation starting after 5 d, effuse on aerial hyphae, pustules formed after 6 d, scattered throughout the colony, hemispherical, compact, 0.5–3 mm, white, turning green after 7 d, hairs extending from the surface of the pustules, straight or sinuous. Chlamydospores rare. No distinct odour, no diffusing pigment observed.

On PDA after 72 h colony radius 62–63 mm, mycelium covering the plate after 4 d at 25 °C. Colony radial, dense, aerial hyphae numerous, long and wooly. Conidiation starting after 2 d, abundant, effuse in aerial hyphae or densely disposed granules, denser at the colony center. Conidiophores simple, asymmetry, verticillium-like, branches often unpaired, not or rebranching once. Phialides solitary, paired or in whorls of 3–4, inclined upwards with the metula, narrowly lageniform, sometimes subulate, (8.9–)10.3–15.8(−17.9) × 2.4–3.3 μm, l/w (2.6–)3.5–5.8(−6.5), 1.9–3.1 μm wide at the base (n = 40). Conidia green, smooth, ellipsoid, less commonly globose, 3.1–4.4 × 2.8–3.3 μm, l/w (1.0–)1.1–1.4 (n = 40). No chlamydospores observed. Light yellow pigment diffusing into the agar. No distinct odour noted.

On SNA after 72 h colony radius 50–51 mm, mycelium covering the plate after 4 d at 25 °C. Colony similar to CMD, but with less aerial hyphae. Conidiation starting after 3 d, effuse on aerial hyphae, pustules formed after 5 d, not common, spreading along the colony margin, hemispherical, 1–4 mm diam, white, turning grayish green after 7 d. Chlamydospores common, globose, ellipsoid or oblong, 4.8–11.0 × 4.5–8.3 μm, l/w 1.0–1.6 (n = 20). No distinct odour, no diffusing pigment observed.

*Additional strain examined*: CHINA, TIBET: Linzhi, Lulang, elev. 3250 m, isolated from soil, September 2016, *K. Chen*, *TC982* (HMAS 248874).

*Notes*: The colony morphology of *T. linzhiense* on PDA is very similar to that of *T. pseudodensum*. Both of them produce dense granules or shrubs on aerial hyphae. In comparison with the new species, *T. pseudodensum* is distinguishable in forming shorter phialides and conidiophores with dense side branches. Phylogenetically, *T. linzhiense* is closely related to *T. cerinum*. But the latter species has much shorter phialides (less than 7.6 μm long) and smaller conidia (2.4–3.5 × 2.0–2.5 μm)^23^.

**Figure 18:**
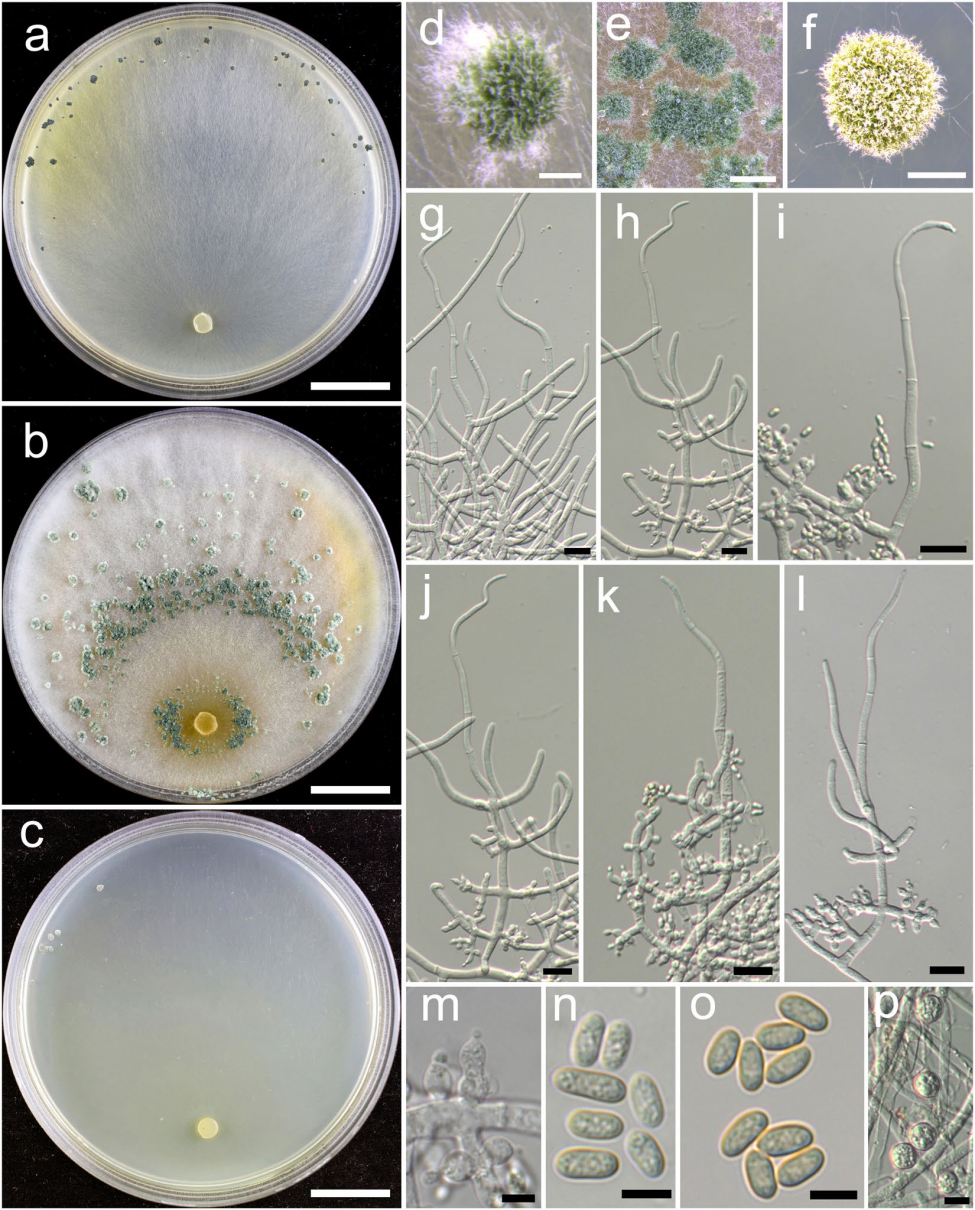
*Trichoderma longisporum* (HMAS 248843). (**a**–**c**) Cultures at 25 °C, 10 d (a. on CMD; b. on PDA; c. on SNA); (**d**,**f**) Conidial pustules (d. CMD, 10 d; e. PDA, 10 d; f. SNA, 10 d); (**g**–**m**) Conidiophores and phialides (PDA, 10 d); (**n**–**o**) Conidia (PDA, 10 d); (**p**) Chlamydospores (PDA, 19 d). Bars: a–c = 20 mm. d = 300 μm. e = 800 μm. f = 600 μm. g–l = 20 μm. m–o = 5 μm. p = 10 μm.

**Trichoderma longisporum** K. Chen & W.Y. Zhuang, **sp. nov**. Figure 18

Fungal Names: FN570393

*Etymology*: The specific epithet refers to the long conidia of the species.

*Holotype*: CHINA, GUANGXI: Chongzuo, Nonggang National Nature Reserve, elev. 300 m, isolated from soil, March 2015, *K. Chen*, *TC673* (HMAS 248843). Ex-type culture CGMCC 3.18397.

On CMD after 72 h colony radius 48–49 mm, mycelium covering the plate after 4 d at 25 °C. Colony hyaline, radial, aerial hyphae nearly lacking. Conidiation noted in pustules after 7 d, pustules appearing around the margin of the colony, hemispherical with irregular outline, 1–2 mm diam, greyish green, with hairs protruding beyond the surface, hairs long, sinuous. No chlamydospores, no distinct odour observed. Light yellowish pigment noted.

On PDA after 72 h colony radius 46–47 mm, mycelium covering the plate after 5 d at 25 °C. Colony radial, not finely zonate, mycelium dense, aerial hyphae short, inconspicuous. Conidiation noted in pustules after 9 d, pustules spreading in concentric areas, hemispherical to pulvinate, outline not well defined, 1–4 mm diam, first white, turning green after 10 d. Conidiophores pachybasium-like, with a distinct main axis, up to 160 μm long, elongations straight or sinuous, tips sterile, fertile braches arising from the base, paired or solitary, not or rebranching once. Phialides ampulliform, 5.6–9.4(−11.4) × 3.6–5.0 μm, l/w 1.2–2.4, 1.9–3.6 μm wide at the base (n = 40). Conidia green, smooth, oblong, (4.4–)5.0–6.4 (−7.5) × 2.6–3.1 μm, l/w (1.6–)1.8–2.3(−2.8) (n = 40). Chlamydospores common, intercalary or terminal, globose to subglobose, (6.6–)8.3–11.7 × (6.6–)7.6–11.0 μm, l/w 1.0–1.3 (n = 33). No distinct odour observed. Light yellowish pigment noted.

On SNA after 72 h colony radius 27–28 mm, mycelium covering the plate after 7 d at 25 °C. Colony hyaline, margin not well defined, aerial hyphae nearly lacking. Conidiation noted in pustules after 10 d, not common, appearing at the colony margin, hemispherical, compact, remaining discrete, 1–2 mm diam, first white, turning green after 11 d, with hairs extending beyond the surface, hairs short, sinuous, sterile at the tips. No chlamydospores, no distinct odour observed. Light yellowish pigment noted.

*Additional strain examined*: CHINA, GUANGXI: Chongzuo, Nonggang National Nature Reserve, elev. 300 m, isolated from soil, September 2016, *K. Chen*, *TC946* (HMAS 248868).

*Notes*: *Trichoderma longisporum* is morphologically similar to *T. oblongisporum*, both species produce relatively large (about 5.0 μm in average length), more or less oblong conidia. But *T. oblongisporum* differs in unbranched and straighter conidiophore elongations^22^. Phylogenetically, *T. longisporum* is closely related to *T. hunanense* and *T. spirale*. The distinctions between *T. hunanense* and *T. longisporum* were already discussed above. *Trichoderma spirale* can be esasily recognized by much shorter phialides (3.3–5.2 × 2.8–4.0 μm) and smaller conidia (3.0–4.4 × 1.8–2.7 μm)^22^.

**Figure 19:**
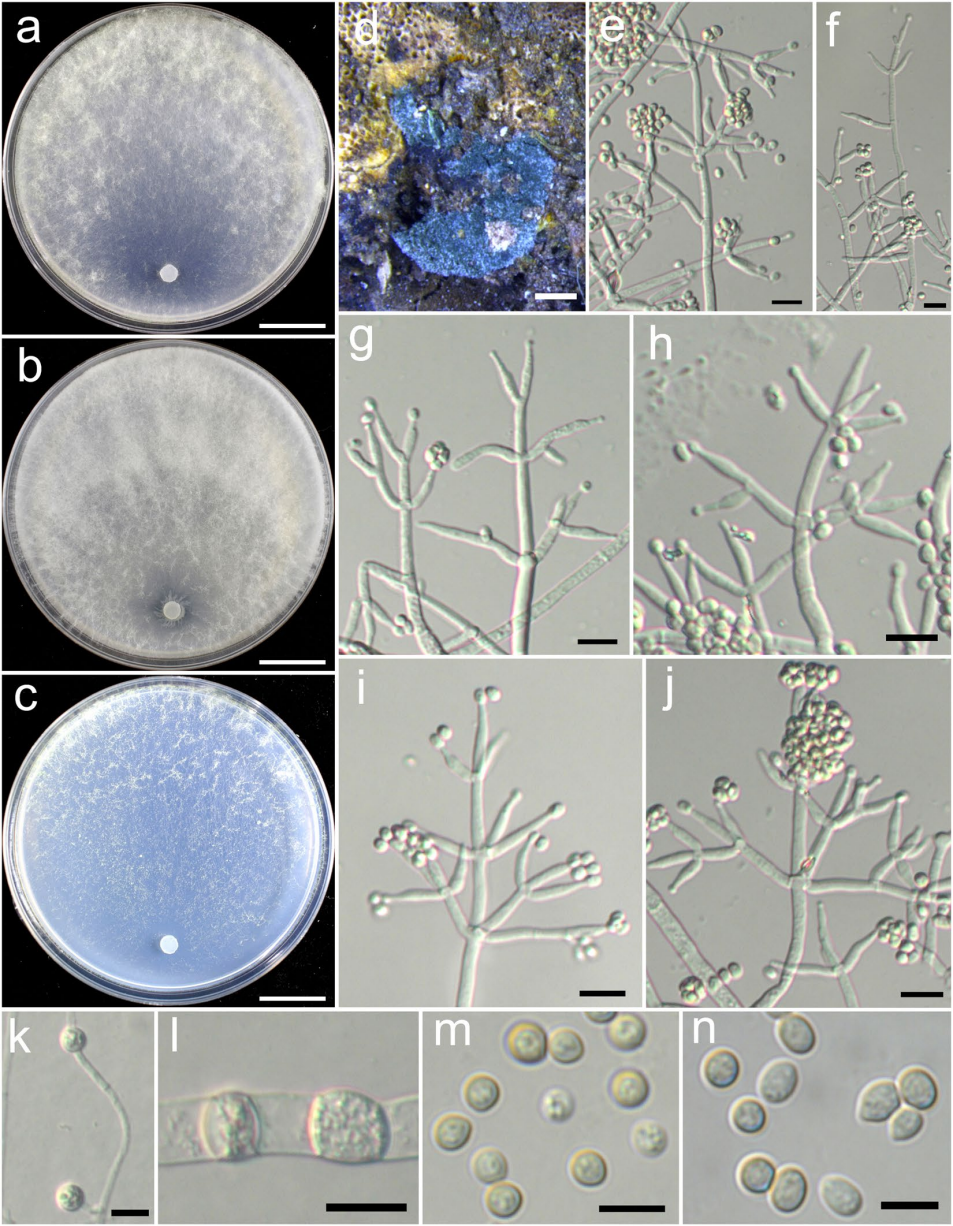
*Trichoderma polypori* (HMAS 248855). (**a**–**c**) Cultures at 25 °C, 7 d (a. on CMD, 10 d; b. on PDA, 7 d; c. on SNA, 7 d); (**d**) Conidial pustules on polypore; (**e**–**j**) Conidiophores and phialides (SNA, 6 d); (**k**,**l**) Chlamydospores (SNA, 6 d); (**m**,**n**) Conidia (SNA, 6 d). Bars: a–c = 20 mm. d = 400 μm. e–l = 10 μm. m–n = 5 μm.

**Trichoderma polypori** K. Chen & W.Y. Zhuang, **sp. nov**. Figure 19

Fungal Names FN570386

*Etymology*: The specific epithet refers to the substrate of the fungus, a dead polypore.

*Holotype*: CHINA, HUNAN: Chenzhou, Mangshan National forest Park, elev. 1700 m, isolated from a dried polypore, October 2015, *K. Chen*,*TC876* (HMAS 248855). Ex-type culture CGMCC 3.18404.

On CMD after 72 h colony radius 44–45 mm, mycelium covering the plate after 4 d at 25 °C. Colony hyaline, mycelium dense, aerial hyphae abundant, floccose, more abundant with distance from the original inoculum. Conidiation starting after 3 d, formed numerous on aerial hyphae. Chlamydospores rare. No distinct odour, no diffusing pigment observed.

On PDA after 72 h colony radius 50–56 mm, mycelium covering the plate after 5 d at 25 °C. Colony similar to CMD, but with more aerial hyphae, coilings common. Conidiation noted after 5 d, rare, on aerial hyphae. Chlamydospores rare. Odour distinctly coconut-like, no diffusing pigment observed.

On SNA after 72 h colony radius 44–49 mm, mycelium covering the plate after 4 d at 25 °C. Colony hyaline, mycelium loose, aerial hyphae common, appearing around the periphery of the colony. Conidiation starting after 3 d, formed on simple conidiophores on aerial hyphae. Conidiophores simple, verticillium-like, short branches arising in acute angles, generally formed in whorls of 3. Phialides formed solitary, paired or in whorls of 3, slender, narrowly lageniform, sometimes subulate, (8.9–)11.7–16.6(−20.0) × 2.4–3.6 μm, l/w (2.4–)3.8–7.0, 1.7–3.1 μm wide at the base (n = 40). Conidia green, smooth, ellipsoid or globose, 2.8–3.6(−4.2) × 2.5–3.3 μm, l/w 1.0–1.4 (n = 40). Chlamydospores numerous, globose or ellipsoid, 5.5–11.7 × 5.5–9.7(−11.0) μm, l/w 1.0–1.2(−1.4) (n = 20). No distinct odour, no diffusing pigment observed.

*Additional strain examined*: CHINA, HUNAN: Chenzhou, Mangshan National forest Park, elev. 1700 m, isolated from a polypore, October 2015, *K. Chen*, *TC908* (HMAS 248861).

*Notes*: *Trichoderma polypori* is isolated from fruitbody of a polypore. It is not clear whether the fungus is mycoparasitic or saprophytic since the polypore was dead and dry when it was found. Phylogenetically, *T. polypori* is related to *T. stramineum* (MPBP/BIPP = 63/98). But *T. stramineum* can be easily distinguished by smaller conidia (3.0–3.2 × 2.0–2.2 μm), pachybasium-like conidiophores and verticillium-like synanamorph^7^.

**Figure 20:**
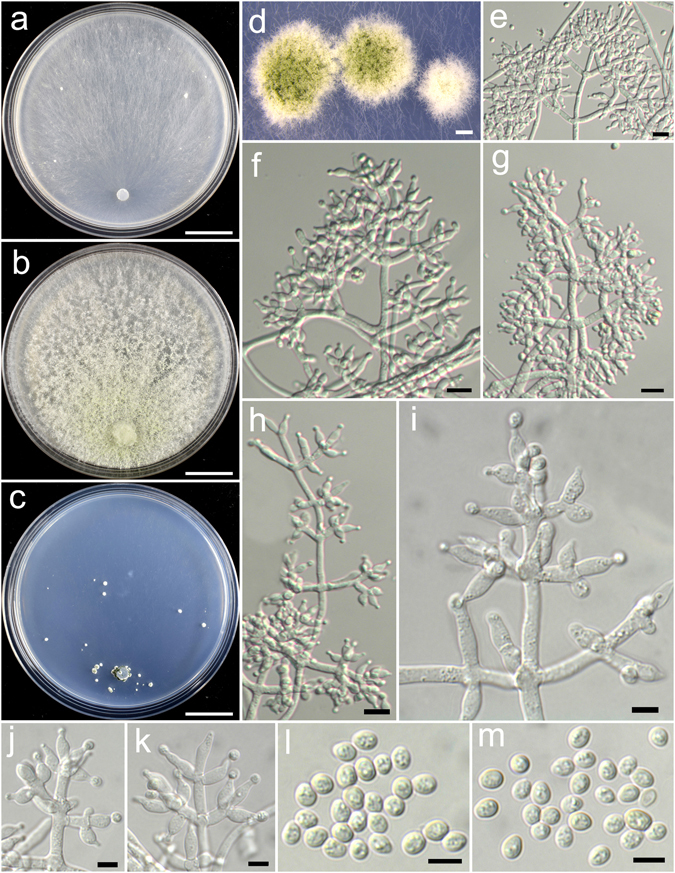
*Trichoderma pseudodensum* (HMAS 248828). (**a**–**c**) Cultures at 25 °C, 7 d (a. on CMD; b. on PDA; c. on SNA); (**d**) Conidial pustules (SNA, 7 d); (**e–k**) Conidiophores and phialides (PDA, 5 d); (**l**,**m**) Conidia (PDA, 5 d). Bars: a–c = 20 mm. d = 400 μm. e–h = 10 μm. i–m = 5 μm.

**Trichoderma pseudodensum** K. Chen & W.Y. Zhuang, **sp. nov**. Figure 20

Fungal Names FN570387

*Etymology*: he specific epithet refers to the densely branched conidiophores.

*Holotype*: CHINA, HUBEI: Shennongjia Natural Reserve, elev. 3100 m, isolated from soil, September 2014, *K. Chen*, *TC222* (HMAS 248828). Ex-type culture CGMCC 3.18387.

On CMD after 72 h colony radius 55–56 mm, mycelium covering the plate after 4 d at 25 °C. Colony hyaline, radial, mycelium dense, aerial hyphae common, denser in distant areas. Conidiation starting after 5 d, first formed on aerial hyphae. Pustules formed after 6 d, rare, hemispherical, 1–2 diam, white, turning green after 7 d. No chlamydospores observed. No distinct odour, no diffusing pigment observed.

On PDA after 72 h colony radius 52–53 mm, mycelium covering the plate after 4 d at 25 °C. Colony radial, dense, circular. Aerial hyphae abundant, forming a flat, dense, whitish mat, coilings common. Conidiation starting after 3 d, effuse in aerial hyphae or in densely disposed granules, more abundant at colony center, yellowish green. Conidiophores numerous, symmetry, trichoderma-like, often with a main axis, side branches densely disposed, in right or acute angles with main axis, typically in whorls of 3, less commonly solitary or paired. Phialides densely disposed, paired or in whorls of 3–4, ampulliform to lageniform, (6.2–)7.6–10.3(−12.4) × 3.3–3.9(−4.2) μm, l/w 2.0–3.0(−4.5), 2.1–2.8 μm wide at the base (n = 40). Conidia light green, smooth, ellipsoid, less commonly globose or oval, 3.1–4.4 × 2.6–3.6 μm, l/w (1.0–)1.1–1.4 (n = 40). No chlamydospores observed. No distinct odour, no diffusing pigment observed.

On SNA after 72 h colony radius 35–38 mm, mycelium covering the plate after 6 d at 25 °C. Colony hyaline, radial, margin not well defined, mycelium loose, aerial hyphae absent. Conidiation starting after 4 d in pustules, pustules common, spreading around the original inoculum, hemispherical, 1–2.5 mm diam, first white, turning grayish green after 6 d. No chlamydospores observed. No distinct odour, no diffusing pigment observed.

*Additional strain examined*: CHINA, HUBEI: Shennongjia Natural Reserve, elev. 3100 m, isolated from soil, September 2014, *K. Chen*, *TC223* (HMAS 248829).

*Notes*: The side branches of conidiophores in *T. pseudodensum* are densely disposed, which is similar to *T. densum* and *T. aggregatum*. But *T. densum* belongs to the Viride Clade, and is phylogenetically distantly related^32^. *Trichoderma aggregatum* produces longer phialides and smaller conidia (see Notes under *T. aggregatum*). Phylogenetically, *T. pseudodensum* is related to *T. zayuense*, but they differ in colony morphology and length of phialides (see also Notes under *T. zayuense*).

**Figure 21:**
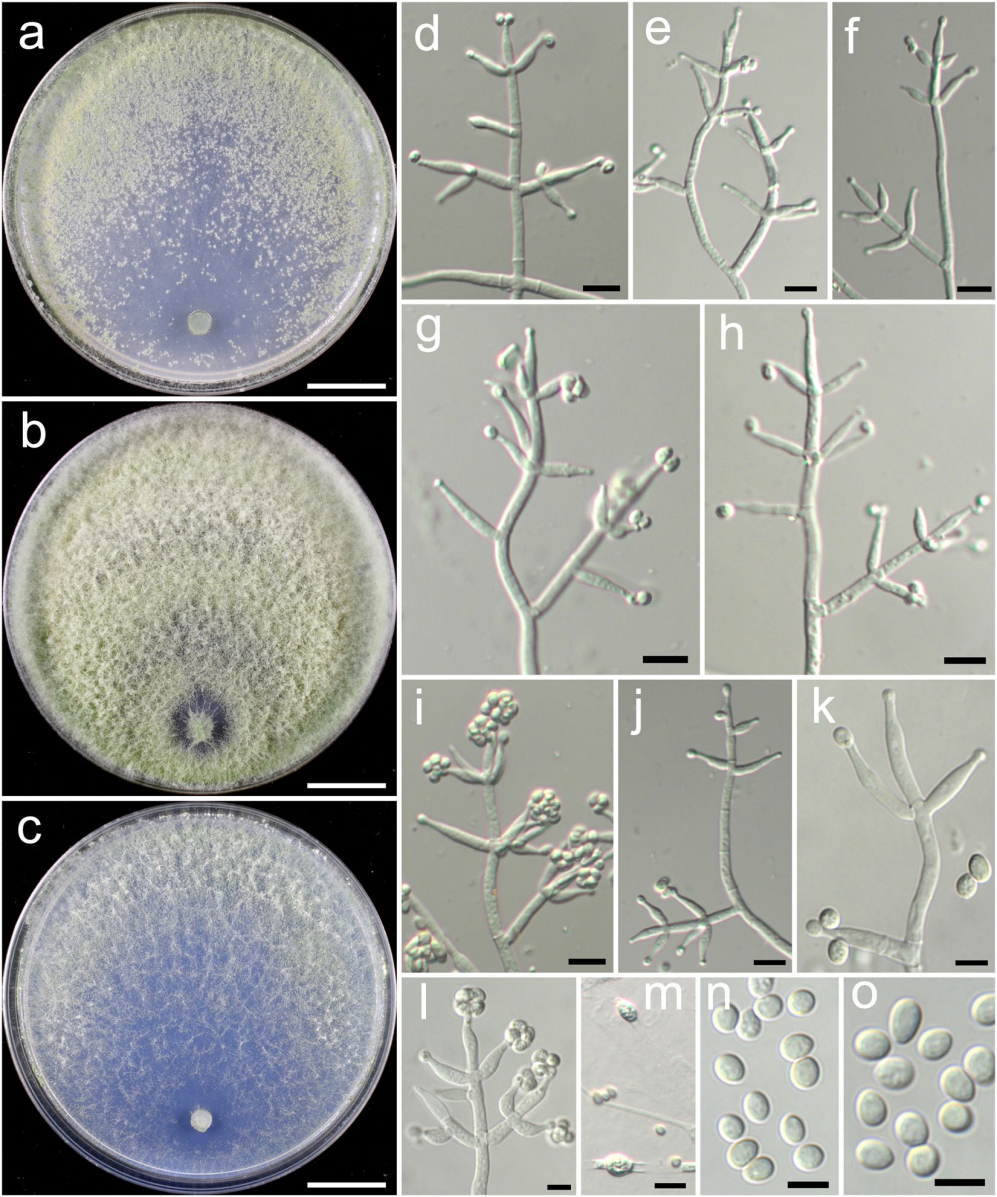
*Trichoderma simplex* (HMAS 248842). (**a**–**c**) Cultures at 25 °C, 7 d (a. on CMD; b. on PDA; c. on SNA); (**d**–**l**) Conidiophores and phialides (PDA, 4 d); (**m**) Chlamydospores (SNA, 7 d); (**n**,**o**) Conidia (PDA, 4 d). Bars: a–c = 20 mm. d–j, m = 10 μm. k, l, n, o = 5 μm.

**Trichoderma simplex** K. Chen & W.Y. Zhuang, **sp. nov**. Figure 21

Fungal Names FN570388

*Etymology*: The specific epithet refers to the simple conidiophores of the species.

*Holotype*: CHINA, GUANGXI: Chongzuo, Nonggang National Nature Reserve, elev. 300 m, isolated from soil, March 2015, *K. Chen*, *TC671* (HMAS 248842). Ex-type culture CGMCC 3.18396.

On CMD after 72 h colony radius 49–56 mm, mycelium covering the plate after 4 d at 25 °C. Colony light green, radial, not zonate, mycelium loose, aerial hyphae common. Conidiation starting after 3 d, effuse in aerial hyphae or in densely disposed loose shrubs, more abundant with distance from the original inoculum. No chlamydospores observed. No distinct odour, no diffusing pigment observed.

On PDA after 72 h colony radius 52–56 mm, mycelium covering the plate after 4 d at 25 °C. Colony circular, conspicuously dense, not finely zonate. Aerial hyphae abundant, long, wooly, coilings common. Conidiation starting after 3 d, numerous, effuse in aerial hyphae. Conidiophores short and simple, verticillium-like, infrequently branched, paired or solitary. Phialides loosely disposed, solitary, paired or in whorls of 3, narrowly lageniform, rarely subulate, straight or curved, (8.6–)10.1–16.9(−19.4) × 2.2–3.6 μm, l/w 2.5–6.7, 1.7–2.9 μm wide at the base (n = 40). Conidia green, smooth, ellipsoid, less commonly globose or oval, 3.1–4.4 × 2.8–3.3 μm, l/w (1.0–)1.1–1.4 (n = 40). No chlamydospores observed. No distinct odour, no diffusing pigment observed.

On SNA after 72 h colony radius 41–44 mm, mycelium covering the plate after 4 d at 25 °C. Colony similar to PDA, but with less aerial hyphae. Conidiation starting after 3 d, formed on aerial hyphae, more abundant in distant areas. Chlamydospores common, globose or ellipsoid, rarely oblong, 4.8–8.9(−10.3) × 4.1–6.9(−8.3) μm, l/w 1.0–1.4(−2.5) (n = 20). No distinct odour, no diffusing pigment observed.

*Additional strain examined*: CHINA, GUANGXI: Chongzuo, Nonggang National Nature Reserve, 300 m, isolated from soil, September 2016, *K. Chen*, *TC907* (HMAS 248860).

*Notes*: *Trichoderma simplex* most resembles *T. hirsutum*, both species produce wooly aerial hyphae on PDA and simple conidiophores. But *T. hirsutum* is distinguished from *T. simplex* in producing chlamydospores on SNA and larger conidia [3.9–4.7(−5.4) × 2.9–3.9 μm]. Phylogenetically, *T. simplex* forms a separate branch related to *T. hausknechtii*, but the latter species grows slower on PDA (35–38 mm after 3 d at 25 °C) and produces shorter phialides (4.3–7.0 × 3.0–3.7 μm)^14^.

**Figure 22:**
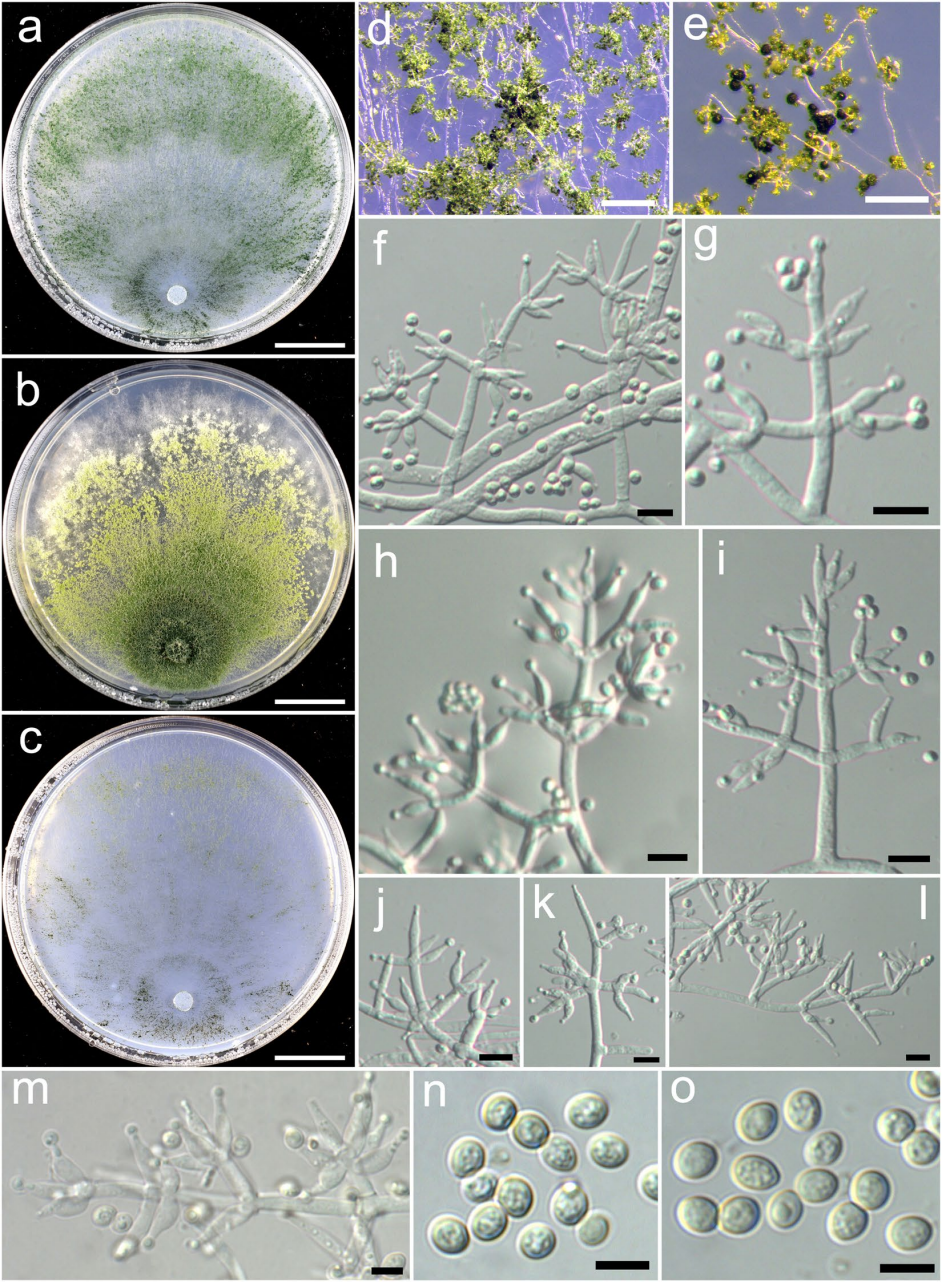
*Trichoderma solum* (HMAS 248848). (**a**–**c**) Cultures at 25 °C, 7 d (a. on CMD; b. on PDA; c. on SNA); (**d**,**e**) Conidial pustules (d. CMD, 7d; e. SNA, 7 d); (**f**–**m**) Conidiophores and phialides (PDA, 3 d); (**n**–**o**) Conidia (PDA, 3 d). Bars: a–c = 20 mm. d = 400 μm. e = 250 μm. f–l = 10 μm. m–o = 5 μm.

**Trichoderma solum** K. Chen & W.Y. Zhuang, **sp. nov**. Figure 22

Fungal Names FN570389

*Etymology*: The specific epithet refers to the phylogeny position of the species which is not closely related to other species in the same clade.

*Holotype*: CHINA, HUBEI: Shennongjia Natural Reserve, elev. 1720 m, isolated from soil, August 2015, *K. Chen*, *TC781* (HMAS 248848). Ex-type culture CGMCC 3.18400.

On CMD after 72 h colony radius 53–56 mm, mycelium covering the plate after 4 d at 25 °C. Colony green, radial, not finely zonate, mycelium loose, aerial hyphae common. Conidiation starting after 2 d, effuse in aerial hyphae or in shrubs, shrubs densely disposed on aerial hyphae, often with green drops. No chlamydospores observed. No distinct odour, no diffusing pigment observed.

On PDA after 72 h colony radius 41–47 mm, mycelium covering the plate after 7 d at 25 °C. Colony green, circular, not zonate, margin not well defined, slightly lobed, substrate mycelium abundant, aerial hyphae common, denser at the colony center. Conidiation starting after 2 d, abundant, formed on aerial hyphae, more abundant at the colony center. Conidiophores short and simple, trichoderma-like, often with a short main axis, side branches in straight or acute angles with the main axis. Phialides formed solitary or in whorls of 3–6, lageniform, less commonly subulate, 8.1–12.5(−14.2) × 2.6–3.9 μm, l/w 2.2–4.6, 1.9–3.1 μm wide at the base (n = 40). Conidia green, smooth, with 1 larger or many minute guttules, ellipsoid or globose, 3.2–4.7 × 3.1–3.6 μm, l/w 1.0–1.5 (n = 40). No chlamydospores observed. No distinct odour, no diffusing pigment observed.

On SNA after 72 h colony radius 40–42 mm, mycelium covering the plate after 5 d at 25 °C. Colony similar to CMD, but with less aerial hyphae and conidiation. No chlamydospores observed. No distinct odour, no diffusing pigment observed.

*Additional strain examined*: CHINA, HUBEI: Shennongjia Natural Reserve, elev. 1720 m, isolated from soil, August 2015, *K. Chen*, *TC778* (HMAS 248847), *TC782* (HMAS 248849).

*Notes*: *Trichoderma solum* is distinguishable by the very dark green granules or tumor-like structures on aerial hyphae, which are uncommon in *Trichoderma*. Phylogenetically, the three strains of *T. solum* with identical sequences form a separate terminal branch which is not close related to any other species in the same clade. *Trichoderma linzhiense* is similar in conidiophore branch patterns and phialides, but differs in colony characteristic, forming yellow pigments on PDA and chlamydospores on CMD and SNA.

**Figure 23:**
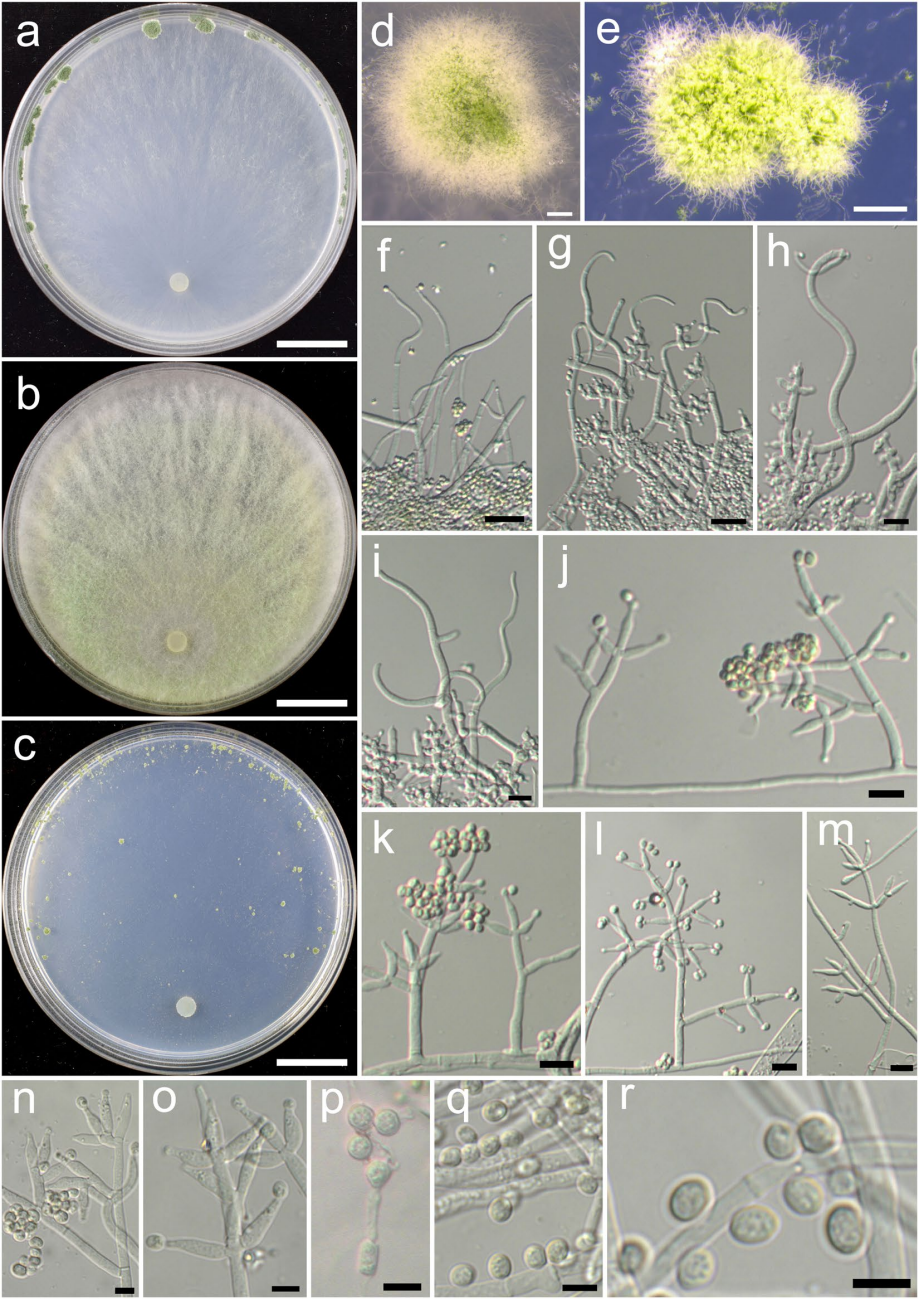
*Trichoderma undatipile* (HMAS 248854). (**a**–**c**) Cultures at 25 °C (a. on CMD, 10 d; b. on PDA, 7 d; c. on SNA, 10 d); (**d**,**e**) Conidial pustules (d. CMD, 7d; e. SNA, 7 d); (**f**–**i**) pachybasium-like conidiophores and phialides (CMD, 7 d); (**j**–**o**) Verticillium-like synanamorph (PDA, 4 d); (**p**) Chlamydospores (SNA, 7 d); (**q**,**r**) Conidia (PDA, 4 d). Bars: a–c = 20 mm. d–e = 200 μm. f–g = 20 μm. h–m, p = 10 μm. n,o,q,r = 5 μm.

**Trichoderma undatipile** K. Chen & W.Y. Zhuang, **sp. nov**. Figure 23

Fungal Names: FN570390

*Etymology*: The specific epithet refers to the curved hairs extending beyond the pustules surface produced in culture.

*Holotype*: CHINA, HUNAN: Hengyang, Hengshan National Nature Reserve, elev. 500 m, isolated from rotten twig, October 2015, *K. Chen*, *TC873* (HMAS 248854). Ex-type culture CGMCC 3.18403.

On CMD after 72 h colony radius 57–59 mm, mycelium covering the plate after 4 d at 25 °C. Colony hyaline, radial, mycelium loose, aerial hyphae inconspicuous. Conidiation noted after 4 d, first formed on short erect conidiophores and aerial hyphae, conidial pustules noted after 5 d, not common, spreading along the margin of the colony, pulvinate, compact, 1–4 mm diam, first white, turning green after 6 d, with hairs extending beyond the surface, hairs curved or sinuous, tips sterile. Conidiophores in pustules pachybasium-like, with a distinct main axis, up to 150 μm, tips sterile, fertile branches arising from the base, short and simple, paired or solitary. Phialides typically formed in whorls of 3–4, ampulliform, 4.8–9.7 × 2.8–3.8 μm, l/w 1.4–2.8, 2.1–3.1 μm wide at the base (n = 30). Conidia green, smooth, mostly ellipsoid, sometimes globose, 2.8–4.7 × 2.5–3.6 μm, l/w 1.0–1.4 (n = 40). Chlamydospores common, terminal or intercalary, typically globose, sometimes ellipsoid or oblong, 4.5–9.0 × 4.5–6.9 μm, l/w 1.0–1.3(−1.9) (n = 25). No distinct odour, no diffusing pigment observed.

On PDA after 72 h colony radius 56–57 mm, mycelium covering the plate after 4 d at 25 °C. Colony radial, mycelium dense, aerial hyphae abundant, spreading uniformly throughout the colony. Conidiation noted after 2 d, formed abundant on aerial hyphae. Conidiophores verticillium-like, short and simple, infrequently branched. Phialides formed solitary, paired or in whorls of 3, narrowly lageniform, sometimes subulate at the terminal position, (8.6–)9.7–13.9(−15.3) × 2.6–3.6 μm, l/w 2.7–5.3, 1.9–3.1 μm wide at the base (n = 30). No chlamydospores observed. No distinct odour, no diffusing pigment observed.

On SNA after 72 h colony radius 37–43 mm, mycelium covering the plate after 5 d at 25 °C. Colony hyaline, mycelium loose, aerial hyphae nearly lacking. Conidial pustules noted after 6 d, common, spreading around the periphery of the colony, hemispherical, 1–2 mm diam, green. Chlamydospores common. No distinct odour, no diffusing pigment observed.

*Additional strain examined*: CHINA, HUNAN: Hengyang, Hengshan National Nature Reserve, elev. 500 m, isolated from rotten twig, October 2015, *K. Chen*, *TC878* (HMAS 248857).

*Notes*: *Trichoderma undatipile* is characterized by its curved or sinuous hairs beyond surface of the pustules in culture. Many *Trichoderma* species form hairs (straight, curved or sinuous) on pustules, e.g. *T. helicum* in the Helicum Clade, *T. longipile* and *T. strictipile* in the Srictipile Clade, and *T. barbatum* in the Stromaticum Clade, etc. But those species are distantly related to *T. undatipile* in the phylogenetic analyses except for *T. helicum*. Compared with the new species, *T. helicum* differs in shorter phialides (2.7–5.8 × 2.5–4.4 μm) and smaller conidia (2.5–3.5 × 1.7–2.5 μm). Besides, verticillium-like synanamorph is noticed in *T. undatipile* while that is absent in *T. helicum* ^23^.

**Figure 24:**
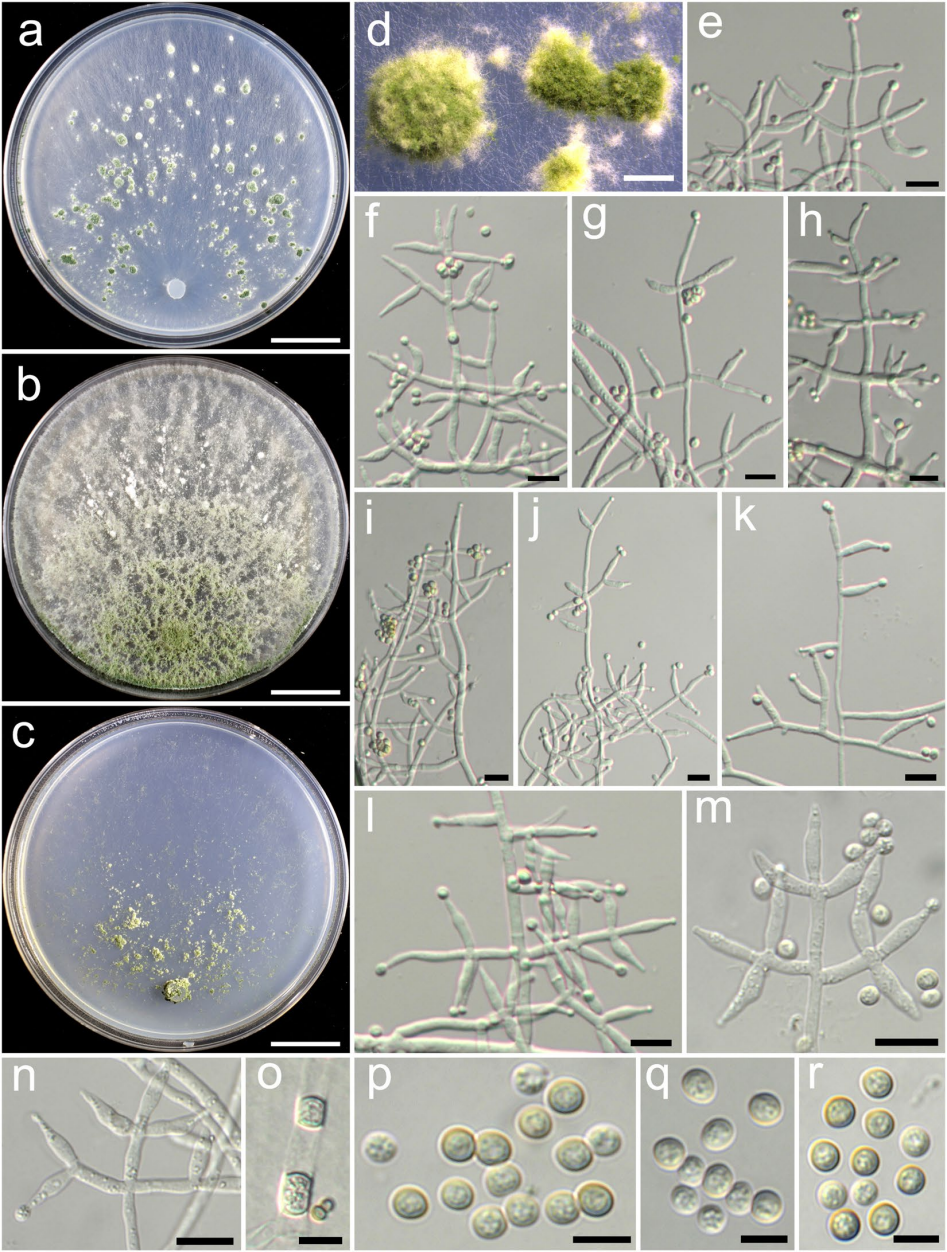
*Trichoderma zayuense* (HMAS 248835). (**a**–**c**) Cultures at 25 °C, 7 d (a. on CMD; b. on PDA; c. on SNA); (**d**) Conidial pustules (CMD, 7 d); (**e**–**n**) Conidiophores and phialides (l. CMD, 6 d; e–g, j, k, m, n. PDA, 6 d; h–i. SNA, 6 d); (**o**) Chlamydospores (SNA, 10 d); (**p**–**r**) Conidia (p. CMD, 6 d; q. PDA, 6 d; r. SNA, 6 d). Bars: a–c = 20 mm. d = 800 μm. e–o = 10 μm. p–r = 5 μm.

**Trichoderma zayuense** K. Chen & W.Y. Zhuang, **sp. nov**. Figure 24

Fungal Names FN570391

*Etymology*: The specific epithet refers to the type locality.

*Holotype*: CHINA, TIBET: Linzhi, Chayu County, elev. 3100 m, isolated from soil, September 2014, *K. Chen*, *TC442* (HMAS 248835). Ex-type culture CGMCC 3.18391.

On CMD after 72 h colony radius 50–51 mm, mycelium covering the plate after 4 d at 25 °C. Colony hyaline, radial, mycelium loose, aerial hyphae inconspicuous. Conidiation starting after 4 d, first on aerial hyphae around the original inoculum, conidial pustules formed after 5 d, spreading in 2–3 inconspicuous concentric rings, denser at colony center, hemispherical, loose, surface downy, with irregular outline, 1–5 mm diam, white, turning green after 6 d. No chlamydospores observed. No distinct odour, no diffusing pigment observed.

On PDA after 72 h colony radius 51–52 mm, mycelium covering the plate after 4 d at 25 °C. Colony radial, not finely zonate, aerial hyphae numerous, long and wooly, more abundant at the colony center. Conidiation starting after 3 d, effuse in aerial hyphae or in densely disposed granules formed on aerial hyphae. Conidiophores symmetry, trichoderma-like, often with a main axis, small side branches disposed evenly along the main axis, often with straight angles, solitary, paired or in whorls of 3. Phialides typically paired, less commonly solitary or in whorls of 3–4, slender, straight or curved, narrowly lageniform, (9.4–)11.1–15.3(−18.6) × 2.2–4.2 μm, l/w 2.8–6.5, 1.8–2.8 μm wide at the base (n = 40). Conidia green, smooth, globose to subglobose, rarely ellipsoid, 3.1–4.2 × 2.8–3.8 μm, l/w 1.0–1.3 (n = 40). Chlamydospores common, globose to subglobose, sometimes oblong, 5.5–7.6(−10.3) × 5.2–6.9 μm, l/w 1.0–1.2(−1.6) (n = 20). No distinct odour, no diffusing pigment observed.

On SNA after 72 h colony radius 32–34 mm, mycelium covering the plate after 5 d at 25 °C. Colony hyaline, margin not well defined, mycelium loose, aerial hyphae inconspicuous. Conidiation starting after 2 d, first on aerial hyphae, conidial pustules noted after 5 d, appearing at the colony center, irregular in shape, 0.5–1 mm diam, first white, turning green after 6 d. Chlamydospores common, No distinct odour, no diffusing pigment observed.

*Additional strain examined*: CHINA, TIBET: Linzhi, Chayu County, elev. 3100 m, isolated from soil, September 2014, *K. Chen*, *TC443* (HMAS 248836).

*Notes*: *Trichoderma zayuense* is featured by its conidiophore branching pattern, in which the side branches are symmetrically disposed at a right angle to the main axis. This pattern is also found in *T. ingratum*, but the conidia of the latter species are smaller (2.6–3.6 × 2.6–3.2 μm) and regularly globose in shape. Phylogenetically, *T. zayuense* is closely related to *T. pseudodensum* but not conspecific (see Notes under *T. pseudodensum*).

## Discussion

### Methods

Elad *et al*.^33^ reported a *Trichoderma* selective medium (TSM) as a tool for isolating *Trichoderma* species from soil. However, the complicated components make TSM unsuitable for isolating soil samples in great numbers. In our study, PDA with chloramphenicol was chosen as the selective medium, and it turns out to be effective. Among the three dilution gradients (10^−1^, 10^−2^ and 10^−3^), 10^−2^ seems to be most appropriate as it forms moderate colonies that can be easily separated from each other.

### Ecology

Geographically, soil samples were mainly collected from mountains or nature reserves in different regions of China, which are relatively undisturbed by humans. Compared with those from farmlands^17, 34–37^, our soil samples show a high species diversity that 23 new species are discovered among the 85 *Trichoderma* species identified. Most of the new species were isolated from southern China (Guangdong, Guangxi, Hainan, Hubei, Hunan, Sichuan, Tibet, and Yunnan). Only two of them (*T. breve* and *T. chlamydosporicum*) were from the north (Beijing and Heilongjiang). It is clear that climates between south and north of the country are significantly different and there are more rains in the south bringing high humidity. This result is in accordance with some earlier studies that southern China are of higher *Trichoderma* species diversity than the northern areas^16, 17^. For example, seven new species were found from Hubei Province in addition to the previously described *T. hubeiense* and *T. shennongjianum* ^18, 38^. Hubei is relatively rich in *Trichoderma* species.

All the new species recorded in this study were isolated from soil except for *T. ganodermatis*, *T. polypori* and *T. undatipile*, which were from fungi or decaying wood. Among soil-inhabiting species, *T. alpinum* and *T. ingratum* are also found on decaying wood (unpublished data). Similarly, *T. ganodermatis* occurs on decaying wood as well. These fungi are obviously not substrate-specific. They may have flexible nutrition modes (fungicolous, saprophytic), or live on different substrata as indicated by Chaverri and Samuels^39^. This makes *Trichoderma* one of the most successful fungi in various ecosystems. The so-called soil-inhibiting species might become well-adopted in diverse environmental conditions.

### Phylogeny

With the addition of the 23 new species, phylogenetic relationships among species of the genus are renewed based on the combined sequence analyses of RPB2 and TEF1. A new clade, the Spirale Clade, is established to accommodate three *Trichoderma* species, *T. hunanense*, *T. longisporum* and *T. spirale*. In the previous studies, phylogenetic position of *T. spirale* was variable. For example, the fungus was reported closely related to *T. polysporum* in the Polysporum clade based on analyses of the combined RPB2 and TEF1^7^. However, the TEF1 phylogeny draws a different picture that it belonged to the Strictipile Clade and was closely related to *T. longipile* and *T. strictipile* ^8^. Jaklitsch and Voglmayr^14^ showed that *T. spirale* was a separate terminal branch, whereas, our results show that *T. spirale* has close relationship with *T. hunanense* and *T. longisporum* receiving very high statistic supports (Fig. 1, MPBP/BIPP = 100%/100%). Fungi in the Spirale Clade share the following morphological similarities: forming hairy pustules, producing yellow pigments in culture, and having more or less oblong conidia.

The Helicum Clade was previously comprised only two species^14^. And the clade appeared to be a separate group distantly related to any other species of the genus. In this study, *T. byssinum*, *T. hainanense* and *T. undatipile* are added. The updated Helicum Clade is associated with *T. pseudonigrovirens* receiving very low statistic supports (Fig. 1).

The Harzianum Clade formerly containing 41 species is the largest among the green-spored groups. As the 15 new species joining the clade, the relationships among members of the clade are altered. *Trichoderma alni* previously related to *T. christiani* ^14^ is now associated with *T. alpinum* (Fig. 1, MPBP/BIPP = 97%/100%); and *T. christiani* is clustered with *T. concentricum*, *T. corneum* and *T. ingratum* with very low supports (Fig. 1). Two of the new species, *T. liberatum* and *T. solum*, form lone lineages which are distantly related to any other species of the clade.

### Future prospects

Identifications of *Trichoderma* species are not always easy. It is impossible to define or recognize a species solely based on morphology, especially when sexual state is absent. Many DNA fragments are available for *Trichoderma* identifications and phylogeny, e.g. ITS, RPB2, TEF1, CAL1 and ACT. ACL1 was recently introduced to study of the genus, which turns out to be efficient^40^. The species concepts may be firmly established with the application of phylogenetic analyses at genomic level.

In summary of the recent studies including this work^11, 12, 18, 32, 38, 41–45^, China is undoubtedly a hotspot of *Trichoderma* species diversity because more than 60 new species have been established and typified by the Chinese materials. However, our knowledge of the group in the country is far from sufficient compared with that in Europe^8, 14, 19^. Further extensive surveys in unexplored areas are surely required.

## Materials and Methods

### Soil samples

A total of 750 soil samples were collected from 23 provinces of China (some were generously provided by colleagues and students) during a three-year-investigation to assess the biodiversity of *Trichoderma* in soil. Most of the samples were from undisturbed areas, e.g. mountains and nature reserves. The soil samples are geographically widely distributed: the northernmost samples are from Heilongjiang Province (53°33′N), and the southernmost are from the Xisha Islands in Hainan Province (15°40′N). The sampled regions comprise diverse climate types: temperate, tropical, subtropical, and alpine climates. The attitudes are ranged from 0 m (Xisha) to 4800 m (Tibet). The various geographic conditions, attitudes and climates makes the samples representative. All the samples were stored at 4 °C or −20 °C before use.

### Strains

Gradient dilution and spread plate method were used to isolate the strains: three dilutions (10^−1^, 10^−2^ and 10^−3^) were prepared with 1 g soil and sterile water, 0.2 mL of each dilution was spread on a 9-cm-diam PDA Petri dish (200 g potato + 2% (w/v) dextrose + 2% (w/v) agar, with 300 mg/L chloramphenicol added). Plates were incubated at 25 °C for 1–3 d. Individual colonies were transferred to a 6-cm-diam PDA Petri dish and cultured at 25 °C. Strains were stored in China General Microbiological Culture Collection Center (CGMCC).

### Morphology observations

Growth rates and culture characteristics were determined on three different media: CMD (40 g cornmeal + 2% (w/v) dextrose + 2% (w/v) agar), PDA and SNA (synthetic low nutrient agar) (Nirenberg 1976). Strains were first cultured on PDA plates for 2–4 d, and then small agar plugs (0.5 cm diam) were cut from actively growing edges of colonies, and placed on 9-cm-diam Petri dishes (CMD, PDA and SNA) 1–1.5 cm away from plate edge. Strains were incubated at 25 °C in 12 h light and 12 h dark. Microscopic structures were observed with 3% KOH solution as mounting medium. Nikon D3300 was used for photographs of colony morphology, and AxioCam MRc5 connected to a Zeiss Imager A2-M2 microscope (Carl Zeiss AG, Göttingen) for microscopic features. Figures were edited and combined using Adobe Photoshop CS5.

### DNA extraction and sequencing

Strains were incubated on PDA plates at 25 °C for 3–7 d. Approximately 1 cm^2^ agar plug with mycelium was cut and transferred to a 1.5 mL Eppendorf tube. Agar plug was grinded with a glass grinding rod and 600 μL CTAB (Hexadecyl trimethyl ammonium bromide) buffer [Tris–HCl 100 mM, EDTA (Ethylenediaminetetraacetic acid) 20 mM, NaCl 1.4 M, CTAB 2% (w/v), β-mercaptoethanol 0.2% (v/v), pH 8.0] was added. Then the Eppendorf tube was incubated at 65 °C for 30 min. Subsequently, 600 μL chloroform-isoamylalcohol (24:1) was added to the solution, mixed and centrifuged for 10 min at 12,000 rpm. Supernatant was transferred to a new tube and 600 μL chloroform-isoamylalcohol (24:1) was added, repeat the previous step. 300 μL isopropanol was added to the supernatant and kept at −20 °C for 30 min. Then the mixture was centrifuged for 10 min at 12,000 rpm and sediment was washed with ice cold 70% ethanol and dried at room temperature. Sediment was re-suspended with 150 μL double distilled water and stored at −20 °C.

The following gene sections were amplified: ITS was amplified using the primer pair ITS 4 and ITS 5^46^; RNA polymerase II subunit B (RPB2) was amplified using the primer pair fRPB2-5f and fRPB2-7cr^47^ or tRPB2-5F and tRPB2-7R^18^; translation elongation factor 1 alpha (TEF1) was amplified using the primers EF1-728F^48^ and TEF1LLErev^49^. PCR products were purified with the PCR product purification kit (Biocolor BioScience & Technology Co., Shanghai, China) and sequenced by the ABI 3730 XL DNA sequencer (Applied Biosystems, Foster City, California) with the same primers as in PCR or internal primers for RPB2 and TEF1^8^.

### Phylogenetic analyses

Sequences were assembled and manually adjusted using the DNAStar Seqman program 7.1.0 (DNASTAR. Inc., Madison). ITS sequences were subjected to TrichOKey^50^ to give a preliminary identification of the *Trichoderma* strains. RPB2 and TEF1 sequences were aligned using ClustalX 1.83^51^. Alignment was desposited in TreeBASE under the submission number 20730. All species of the related taxa were included in the analyses. *Trichoderma rossicum* and *T. stromaticum* in the Stromaticum Clade were chosen as outgroup taxa. Sequences used in the analyses are listed in Supplementary Table S1.

MP analyses were performed with PAUP* 4.0b 10^52^, using 1000 replicates of heuristic search with random addition of sequences and tree bisection-reconnection (TBR) as the branch-swapping algorithm (steepest descent option not in effect, MULTREES option not in effect). All characters were weighted equally and gaps were treated as missing characters. Maximum parsimony bootstrap proportions (MPBP) were calculated from 1000 replicates, each with 10 replicates of random addition of taxa.

For BI analyses, MrBayes 3.1.2^53^ was used and the substitution model for each locus was estimated by MrModeltest 2.3^54^. HKY + I + G was selected as the best-fit likelihood model under the output strategy of Akaike Information Criterion (AIC). Metropolis-coupled Markov chain Monte Carlo (MCMCMC) searches were run for 2000000 generations sampling every 100 generation. The first 2500 trees were discarded as the burn-in phase, and Bayesian inference posterior probability (BIPP) was determined from the remaining trees.

Trees were viewed and examined by TreeView 1.6.6^55^ with MPBP above 50% and BIPP above 90% shown at the nodes.

## Electronic supplementary material

Supplemental Information
