# Supplementary material for: Discovery from a large-scaled survey of Trichoderma in soil of China
Source: Sci Rep. 2017 Aug 22;7:9090. doi: 10.1038/s41598-017-07807-3 (PMC5567330; doi:10.1038/s41598-017-07807-3)
Supplement: Supplementary file 1 — Supplemental Information [file 41598_2017_7807_MOESM1_ESM.pdf]

Discovery from a large-scaled survey of *Trichoderma* in soil of China

Kai Chen[1, 2], Wen-Ying Zhuang[1, 2*]

[1] State Key Laboratory of Mycology, Institute of Microbiology, Chinese Academy of Sciences, Beijing 100101, China

[2] University of Chinese Academy of Sciences, Beijing 100049, China

[*]Corresponding author: E-mail: zhuangwy@im.ac.cn

Supplementary Table S1 Sequences used in this study

Species	Strains	GenBank accession No.		
		ITS	RPB2	TEF1
Trichoderma aerugineum Jaklitsch	CBS 120541		FJ860516	FJ860608
T. afarasin P. Chaverri & Branco-Rocha	DIS 377A		FJ442799	FJ463322
T. afroharzianum P. Chaverri, F.B. Rocha & I. Druzhinina	GJS 04-186		FJ442691	FJ463301
T. aggregatum K. Chen & W.Y. Zhuang	HMAS 248863	**KY687946**	**KY688001**	**KY688062**
	HMAS 248864	**KY687947**	**KY688002**	**KY688063**
T. aggressivum Samuels & W. Gams	CBS 100526		KP009166	KP008993
T. alni Jaklitsch	CBS 120633		EU498349	EU498312
	CPK 2494		EU498350	EU498313
T. alpinum K. Chen & W.Y. Zhuang	HMAS 248821	**KY687906**	**KY687958**	**KY688012**
	HMAS 248823	**KY687904**	**KY687959**	**KY688013**
	HMAS 248825	**KY687907**	**KY687960**	**KY688014**
	HMAS 248830	**KY687912**	**KY687961**	**KY688015**
	HMAS 248862	**KY687945**	**KY687962**	**KY688016**
	HMAS 248870	**KY687953**	**KY687963**	**KY688017**
T. amazonicum P. Chaverri & Gazis	IB95		HM142368	HM142377
T. atrobrunneum F.B. Rocha, P. Chaverri & Jaklitsch	CPK 1934		FJ179608	FJ179573
T. atrogelatinosum (Dingley) Jaklitsch & Voglmayr	LU498		KJ842176	KJ871087
T. attinorum Q.V. Montoya, L.A. Meirelles, P. Chaverri & A. Rodrigues	LESF 236		KT278971	KT279039
T. aureoviride Rifai	CPK 2848		JQ685882	FJ860615
T. bennaense K. Chen & W.Y. Zhuang	HMAS 248840	**KY687923**	**KY687979**	**KY688037**
	HMAS 248865	**KY687948**	**KY688003**	**KY688038**
T. breve K. Chen & W.Y. Zhuang	HMAS 248844	**KY687927**	**KY687983**	**KY688045**
	HMAS 248845	**KY687928**	**KY687984**	**KY688046**
T. brevicrassum K. Chen & W.Y. Zhuang	HMAS 248871	**KY687954**	**KY688008**	**KY688064**
	HMAS 248872	**KY687955**	**KY688009**	**KY688065**
T. britannicum (Rifai & J. Webster) Jaklitsch & Voglmayr	SB1		KF134787	KF134796

T. brunneoviride Jaklitsch	CBS 121130		–	EU498316
T. byssinum K. Chen & W.Y. Zhuang	HMAS 248838	**KY687921**	**KY687977**	**KY688035**
	HMAS 248839	**KY687922**	**KY687978**	**KY688036**
T. camerunense P. Chaverri & G.J. Samuels	GJS 99-230		–	AF348107
T. catoptron P. Chaverri & Samuels	GJS 02-76		AY391900	AY391963
T. ceraceum P. Chaverri & Samuels	GJS 95-159		AY391901	AY391964
T. ceramicum P. Chaverri & Samuels	CBS 114576		FJ860531	FJ860628
T. cerinum Bissett, C.P. Kubicek & Szakács	DAOM 230012		KJ842184	AY605802
	S357		KF134788	KF134797
T. chlamydosporicum K. Chen & W.Y. Zhuang	HMAS 248850	**KY687933**	**KY687989**	**KY688052**
	HMAS 248851	**KY687934**	**KY687990**	**KY688053**
T. chlorosporum P. Chaverri & Samuels	GJS 88-33		AY391903	AY391966
T. christiani Jaklitsch & Voglmayr	S43		KJ665243	KJ665438
	S442		KJ665244	KJ665439
T. chromospermum P. Chaverri & Samuels	GJS 94-68		AY391913	AY391974
T. cinnamomeum P. Chaverri & Samuels	GJS 97-237		AY391920	AY391979
T. compactum Z.F. Yu & K.Q. Zhang	CBS 121218		KF134789	KF134798
T. concentricum K. Chen & W.Y. Zhuang	HMAS 248833	**KY687915**	**KY687971**[*]	**KY688027**
	HMAS 248858	**KY687941**	**KY687997**	**KY688028**
T. corneum (Pat.) Jaklitsch & Voglmayr	GJS 97-82		KJ665252	KJ665455
T. costaricense (P. Chaverri & Samuels) P. Chaverri, Jaklitsch & Voglmayr	PC 21		AY391921	AY391980
T. crissum Bissett	DAOM 164916		AF545542	AF534615
T. cremeoides Jaklitsch & Voglmayr	S112		KF134790	KJ665456
T. cremeum P. Chaverri & Samuels	GJS 91-125		AF545511	AF534598
T. dacrymycellum Jaklitsch	WU 29044		FJ860533	FJ860633
T. danicum (Jaklitsch) Jaklitsch & Voglmayr	CBS 121273		FJ860634	FJ860534
T. endophyticum F.B. Rocha, Samuels & P. Chaverri	DIS 217H		FJ442721	FJ463314
T. epimyces Jaklitsch	CBS 120534		EU498360	EU498320
T. estonicum P. Chaverri & Samuels	GJS 96-129		AF545514	AF534604
T. ganodermatis K. Chen & W.Y. Zhuang	HMAS 248856	**KY687939**	**KY687995**	**KY688060**
	HMAS 248869	**KY687952**	**KY688007**	**KY688061**
T. gelatinosum P. Chaverri & Samuels	GJS 88-17		AF545516	AF534579
T. gliocladium Jaklitsch & Voglmayr	S81		KJ665271	KJ665502
T. guizhouense Q.R. Li, McKenzie & Yong Wang bis	CBS 131803		JQ901401	JX089585
T. hainanense K. Chen & W.Y. Zhuang	HMAS 248837	**KY687920**	**KY687976**	**KY688033**
	HMAS 248866	**KY687949**	**KY688004**	**KY688034**
T. harzianum Rifai	CBS 226.95		AF545549	AF348101
T. hausknechtii Jaklitsch & Voglmayr	Hypo 649		KJ665276	KJ665515
T. helicolixii Jaklitsch & Voglmayr	S640		KJ665278	KJ665517
T. helicum Bissett, C.P. Kubicek & Szakács	DAOM 230021		DQ087239	KJ871125
T. hengshanicum K. Chen & W.Y. Zhuang	HMAS 248852	**KY687935**	**KY687991**	**KY688054**

	HMAS 248853	**KY687936**	**KY687992**	**KY688055**
T. hirsutum K. Chen & W.Y. Zhuang	HMAS 248834	**KY687916**	**KY687972**	**KY688029**
	HMAS 248859	**KY687942**	**KY687998**	**KY688030**
T. hunanense K. Chen & W.Y. Zhuang	HMAS 248841	**KY687924**	**KY687980**	**KY688039**
	HMAS 248867	**KY687950**	**KY688005**	**KY688040**
T. ingratum K. Chen & W.Y. Zhuang	HMAS 248822	**KY687917**	**KY687973**	**KY688018**
	HMAS 248824	**KY687905**	**KY687964**	**KY688019**
	HMAS 248826	**KY687908**	**KY687965**	**KY688020**
	HMAS 248827	**KY687909**	**KY687966**	**KY688021**
	HMAS 248873	**KY687956**	**KY688010**	**KY688022**
T. inhamatum Veerkamp & W. Gams	CBS 273.78		FJ442725	AF348099
T. italicum Jaklitsch & Voglmayr	S131		KJ665282	KJ665525
T. lentiforme (Rehm) P. Chaverri, Samuels & F.B. Rocha	DIS 94D		FJ442749	FJ463379
T. liberatum K. Chen & W.Y. Zhuang	HMAS 248831	**KY687913**	**KY687969**	**KY688025**
	HMAS 248832	**KY687914**	**KY687970**	**KY688026**
T. linzhiense K. Chen & W.Y. Zhuang	HMAS 248846	**KY687929**	**KY687985**	**KY688047**
	HMAS 248874	**KY687957**	**KY688011**	**KY688048**
T. lixii (Pat.) P. Chaverri	CBS 110080		KJ665290	FJ716622
T. longifialidicum	LESF 552		KT278955	KT279020
T. longipile Bissett	CBS 120953		FJ860542	FJ860643
T. longisporum K. Chen & W.Y. Zhuang	HMAS 248843	**KY687926**	**KY687982**	**KY688043**
	HMAS 248868	**KY687951**	**KY688006**	**KY688044**
T. lycogaloides (Berk. & Broome) Jaklitsch, Lechat & Voglmayr	SL		KF134792	KF134800
T. neotropicale P. Chaverri & F. Branco-Rocha	LA11		–	HQ022771
T. parepimyces Jaklitsch	CBS 122769		FJ860562	FJ860664
T. parestonicum Jaklitsch	CBS 120636		FJ860565	FJ860667
T. phyllostachydis P. Chaverri & Samuels	CBS114071		FJ860570	FJ860673
T. pleuroti S.H. Yu & M.S. Park	CBS124387		HM142372	HM142382
T. pleuroticola S.H. Yu & M.S. Park	CBS124383		HM142371	HM142381
T. polypori K. Chen & W.Y. Zhuang	HMAS 248855	**KY687938**	**KY687994**	**KY688058**
	HMAS 248861	**KY687944**	**KY688000**	**KY688059**
T. priscilae Jaklitsch & Voglmayr	S168		KJ665333	KJ665691
T. pseudocandidum Minnis, Samuels & P. Chaverri	PC 59		AY391899	AY391962
T. pseudodensum K. Chen & W.Y. Zhuang	HMAS 248828	**KY687910**	**KY687967**	**KY688023**
	HMAS 248829	**KY687911**	**KY687968**	**KY688024**
T. pseudogelatinosum (M. Komatsu & Yoshim. Doi) C.S. Kim	CNUN309		HM920173	HM920202
T. pseudonigrovirens Minnis, Samuels & P. Chaverri	GJS 99-64		AF545518	AF534582
T. pyramidale Jaklitsch & P. Chaverri	S73		KJ665334	KJ665699
T. rifaii F.B. Rocha, P. Chaverri & Samuels	DIS 337F		FJ442720	FJ463321

T. rossicum Bissett, C.P. Kubicek & Szakács	DAOM 230010		HQ400747	AY937424
T. rosulatum Z.X. Zhu & W.Y. Zhuang	HMAS 252548		KF730005	KF729984
T. rufobrunneum Z.X. Zhu & W.Y. Zhuang	HMAS 266614		KF730010	KF729989
T. shennongjianum K. Chen & W.Y. Zhuang	HMAS 245009		KT735259	KT735253
T. silvae-virgineae Jaklitsch	CBS 120922		FJ860587	FJ860696
T. simmonsii P. Chaverri, F.B. Rocha, Samuels & W. Jaklitsch	GJS 91-138		FJ442757	AF443935
T. simplex K. Chen & W.Y. Zhuang	HMAS 248842	**KY687925**	**KY687981**	**KY688041**
	HMAS 248860	**KY687943**	**KY687999**	**KY688042**
T. sinuosum P. Chaverri & Samuels	PC 8		–	AY391997
T. solum K. Chen & W.Y. Zhuang	HMAS 248847	**KY687930**	**KY687986**	**KY688049**
	HMAS 248848	**KY687931**	**KY687987**	**KY688050**
	HMAS 248849	**KY687932**	**KY687988**	**KY688051**
T. spinulosum (Fuckel) Jaklitsch & Voglmayr	CBS 121280		FJ860589	FJ860699
T. spirale Bissett	DAOM 183974		AF545553	AF534626
T. stipitatum Z.X. Zhu & W.Y. Zhuang	HMAS 266612		KF730011	KF729990
T. stramineum P. Chaverri & Samuels	GJS 02-84		AY391945	AY391999
T. strictipile Bissett	CPK 1601		FJ860594	FJ860704
T. stromaticum Samuels & Pardo-Schulth	GJS 97-180		HQ342226	HQ342166
T. sulawesense (Yoshim. Doi) Jaklitsch & Voglmayr	GJS 85-228		AY391954	AY392002
T. surrotundum P. Chaverri & Samuels	GJS 88-73		AF545540	AY737734
T. tawa P. Chaverri & Samuels	GJS 97-174		AY391956	AY392004
T. thailandicum P. Chaverri & Samuels	GJS 97-61		AY391957	AY392005
T. thelephoricola P. Chaverri & Samuels	CBS 120925		JQ685886	FJ860711
T. tibetense K. Chen & W.Y. Zhuang	HMAS 245010		KT735261	KT735254
T. tomentosum Bissett	DAOM 178713a		AF545557	AF534630
T. tropicosinense (P.G. Liu) Z.X. Zhu & W.Y. Zhuang	HMAS 252546		KF923313	KF923286
T. undatipile K. Chen & W.Y. Zhuang	HMAS 248854	**KY687937**	**KY687993**	**KY688056**
	HMAS 248857	**KY687940**	**KY687996**	**KY688057**
T. velutinum Bissett, C.P. Kubicek & Szakács	CPK 298		KF134794	KJ665769
T. virens (J.H. Mill., Giddens & A.A. Foster) Arx	Gli 39		AF545558	AF534631
T. virescentiflavum (Speg.) Jaklitsch & Voglmayr	PC 278		AY391959	AY737749
T. zayuense K. Chen & W.Y. Zhuang	HMAS 248835	**KY687918**	**KY687974**	**KY688031**
	HMAS 248836	**KY687919**	**KY687975**	**KY688032**

[*]Numbers in boldface indicate newly submitted sequences.
